# Elevated expression of glycolytic genes as a prominent feature of early-onset preeclampsia: insights from integrative transcriptomic analysis

**DOI:** 10.3389/fmolb.2023.1248771

**Published:** 2023-09-25

**Authors:** Jie He, Huan Yang, Zheng Liu, Miaomiao Chen, Ying Ye, Yuelan Tao, Shuhong Li, Jie Fang, Jiacheng Xu, Xiafei Wu, Hongbo Qi

**Affiliations:** ^1^ Department of Obstetrics, The First Affiliated Hospital of Chongqing Medical University, Chongqing, China; ^2^ Chongqing Key Laboratory of Maternal and Fetal Medicine, Chongqing Medical University, Chongqing, China; ^3^ Joint International Research Laboratory of Reproduction and Development of Chinese Ministry of Education, Chongqing Medical University, Chongqing, China; ^4^ Department of Obstetrics, Chongqing University Three Gorges Hospital, Chongqing, China; ^5^ Maternal and Child Health Hospital of Hubei Province, Wuhan, China; ^6^ Department of Cardiothoracic Surgery, The Second Affiliated Hospital of Chongqing Medical University, Chongqing, China; ^7^ Department of Oncology, Chengdu Second People’s Hospital, Chengdu, China; ^8^ Department of Obstetrics and Gynecology, Women and Children’s Hospital of Chongqing Medical University, Chongqing, China

**Keywords:** preeclampsia, placenta, gestational hypertension, transcriptomics, microarray, single-cell sequencing

## Abstract

**Introduction:** Preeclampsia (PE), a notable pregnancy-related disorder, leads to 40,000+ maternal deaths yearly. Recent research shows PE divides into early-onset (EOPE) and late-onset (LOPE) subtypes, each with distinct clinical features and outcomes. However, the molecular characteristics of various subtypes are currently subject to debate and are not consistent.

**Methods:** We integrated transcriptomic expression data from a total of 372 placental samples across 8 publicly available databases via combat algorithm. Then, a variety of strategies including Random Forest Recursive Feature Elimination (RF-RFE), differential analysis, oposSOM, and Weighted Correlation Network Analysis were employed to identify the characteristic genes of the EOPE and LOPE subtypes. Finally, we conducted in vitro experiments on the key gene HK2 in HTR8/SVneo cells to explore its function.

**Results:** Our results revealed a complex classification of PE placental samples, wherein EOPE manifests as a highly homogeneous sample group characterized by hypoxia and HIF1A activation. Among the core features is the upregulation of glycolysis-related genes, particularly HK2, in the placenta-an observation corroborated by independent validation data and single-cell data. Building on the pronounced correlation between HK2 and EOPE, we conducted in vitro experiments to assess the potential functional impact of HK2 on trophoblast cells. Additionally, the LOPE samples exhibit strong heterogeneity and lack distinct features, suggesting a complex molecular makeup for this subtype. Unsupervised clustering analysis indicates that LOPE likely comprises at least two distinct subtypes, linked to cell-environment interaction and cytokine and protein modification functionalities.

**Discussion:** In summary, these findings elucidate potential mechanistic differences between the two PE subtypes, lend support to the hypothesis of classifying PE based on gestational weeks, and emphasize the potential significant role of glycolysis-related genes, especially HK2 in EOPE.

## Introduction

Preeclampsia (PE) is a common multi-organ pregnancy complication characterized by the development of hypertension and proteinuria after 20 weeks of gestation. In severe cases, it may progress to neurological symptoms such as seizures, which are collectively known as eclampsia, hence the term “preeclampsia” (Magee et al., 2022). PE affects 2%–4% of pregnant women worldwide and is a leading risk factor for perinatal mortality, resulting in an estimated 500,000 perinatal deaths each year (Magee et al., 2022). Consequently, it is an essential focal point of obstetrical research. Before delivery at 34 weeks, obstetricians classify pregnant women with pre-eclampsia into two subtypes: early-onset PE (EOPE) and late-onset PE (LOPE) (Jung et al., 2022). Patients with EOPE typically exhibit more severe clinical symptoms and a poorer prognosis, suggesting potential differences in pathogenic mechanisms between the two subtypes (Jung et al., 2022). This classification not only facilitates distinct clinical management approaches but also enables a deeper understanding of the underlying mechanisms of pre-eclampsia. According to accumulated research results, a widely accepted theory suggests that the pathogenesis of EOPE is primarily due to placental formation disorders that occur during early pregnancy (Gj et al., 2019). Conversely, LOPE is believed to be caused primarily by an increase in fetal demand (Gj et al., 2019). Both mechanisms arise from an absolute and relative mismatch between placental function and fetal demand, leading to stress in the placental trophoblast and the release of stress-related factors (Gj et al., 2019). This can cause dysfunction and inflammation of the vasculature in multiple organ systems, resulting in the characteristic multi-organ damage associated with pre-eclampsia. To recap, placental serves as the cornerstone of pre-eclampsia pathogenesis, and as such, delivery remains the only effective treatment for this condition.

Given the essential role of placenta in the pathogenesis of pre-eclampsia (PE), extensive research has been conducted on PE placenta to understand the underlying molecular mechanisms (Cruz-Lemini et al., 2022; Louwen et al., 2022; Melchiorre et al., 2022). Transcriptome data, which are the predominant type of research data (Szilagyi et al., 2020; Yadama et al., 2020; Ren et al., 2021), have been shared in databases for further exploration. However, the differences in data quality, batch, and sample subtypes have become significant challenges for utilizing this data (Lazar et al., 2013). Firstly, some independent studies have insufficiently recognized the potential mechanistic differences between EOPE and LOPE, and this, coupled with limited sample sizes and a lack of differentiation in disease subtypes within the samples, compromises the generalizability of research outcomes. For example, two similar studies (each with sample sizes below 10) arrived at different conclusions, identifying angiogenesis-associated genes and inflammatory response-associated genes as distinct elements in EOPE and LOPE samples (Junus et al., 2012; Liang et al., 2016). This alludes to the probability that these conclusions predominantly echo the attributes of the specific samples under consideration, rather than holding broader applicability. Furthermore, evident batch effects traverse across various datasets, intricately interwoven with biological variabilities across samples. Thoughtfully designed algorithms are imperative to ameliorate batch effects prior to embarking on integrated analyses. The intricacies inherent in these research findings and the methodological challenges at hand currently obstruct a holistic comprehension of the pathogenesis of PE.

In order to fully capitalize on the transcriptomic data of EOPE and LOPE placentas, we integrated eight microarray-based datasets comprising a total of 372 human placenta samples. We then employed machine learning techniques, including random forest and artificial neural networks, to extract valuable insights from these datasets. To overcome the challenges posed by batch effects on data integration, we turned to the Combat software for batch correction, and our analytical outcomes were also confirmed across multiple datasets. Combat is a wildly-used and dependable statistical model that rectifies batch-induced differences while preserving authentic variability across distinct batches (Leek et al., 2012). Traditional transcriptomic analysis methods rely on manually selecting threshold-based differential gene analysis approaches, which possess a degree of subjectivity. In order to sidestep biases introduced by adjustable threshold parameters in traditional differential gene analysis, we utilized the oposSOM software, an unsupervised neural network approach that extracted readable molecular pathological features from the high-dimensional gene expression matrix (Löffler-Wirth et al., 2015).

From our analysis of the combined dataset, we observed a strong homogeneity in the expression of PE-related genes in EOPE placentas, whereas LOPE placentas displayed greater heterogeneity, highlighting the complexity of classifying PE placentas. Subsequent analysis enabled us to identify the molecular expression patterns associated with EOPE and reveal that genes involved in the glycolysis pathway, such as HK2, TPI1, SLC2A1, were significantly upregulated in EOPE placenta, which was further substantiated in a single-cell sequencing dataset. Among the examined genes, HK2 manifests the most pronounced differential fold change and displays a notable correlation with maternal hypertension. To elucidate the plausible role of HK2 within the placenta, a series of *in vitro* experiments were meticulously conducted employing trophoblast cells. In addition, unsupervised clustering algorithms identified two distinct potential molecular subtypes within the heterogeneous LOPE placental samples, indicating the diversity of mechanisms underlying LOPE.

## Materials and methods

### Data retrieval and grouping

Microarray datasets for integration were obtained from the public database Gene Expression Omnibus (Gene Expression Omnibus, 2023) (www.ncbi.nlm.nih.gov/geo/) with the following reference numbers: GSE14722, GSE22526, GSE25906, GSE35574, GSE44711, GSE66273, GSE74341, and GSE75010. Based on the clinical gestational age or sample grouping information of each placenta sample, the samples were reclassified into four groups: EOPE, LOPE, preterm, and term. Placental samples from PE patients with a clinical gestational age <34 weeks were classified as EOPE, those with 34≤ clinical gestational age <37 weeks were classified as LOPE; non-PE samples with a clinical gestational age <37 weeks were classified as preterm, and non-PE samples with a clinical gestational age ≥37 weeks were classified as term. Furthermore, for the validation of gene expression levels in the integrated matrix, we utilized the dataset with the accession number GSE148241. Single-cell placental data from PE patients originated from the dataset GSE173193.

### Microarray raw data processing and quality control

Microarray raw data was downloaded from the GEO database. Since the included datasets were from different experimental platforms, we chose methods suitable for each chip’s experimental design for quality control. Specifically, for dual-channel chips, the median signal of the sample channel after background correction in the sample channel was used as the probe fluorescence reading, and probes with readings <5 were removed. For single-channel chips with background correction, probes with a fluorescence-to-background comparison *p*-value ≥0.05 were removed. For CEL format single-channel raw data, the “affy” package (Gautier et al., 2004) pipeline was used for processing, and the “genefilter” package (Robert, 2023) was used to remove probes with low variability. For probe sets in the raw data that come with sample probe detection *p*-values, probes with *p*-values ≥ 0.05 were filtered out. All fluorescence readings corrected and quality-controlled were log2 transformed, and probes were annotated based on the chip’s specifications.

### Batch correction

Batch correction was performed using the “Combat” package (Leek et al., 2012). First, 4,816 genes shared by the eight matrices to be merged were extracted for merging, and different studies were designated as different batches. Specifically, the GSE35574 and GSE255906 datasets themselves have two batches. Therefore, the eight expression matrices were designated as ten batches. The linear regression model was used to describe the sample subtype grouping, and the Combat function mod parameter was used for matrix batch effect correction. The batch effect-corrected matrix was used for further analysis.

### Random forest recursive feature elimination (RF-RFE)

The recursive feature elimination (RFE) function of the “caret” package (Kuhn, 2008) was used with 10-fold cross-validation as the algorithm’s cross-validation method, and the accuracy of feature set sizes ranging from 1 to 100 and from 200 to 3,200 in an exponential sequence was calculated to determine the optimal feature subset.

### Differential analysis and gene ontology (GO) enrichment analysis

The “limma” package (Ritchie et al., 2015) was used for differential analysis, which involved fitting a linear model to the expression matrix, calculating contrast coefficients, and estimating the statistical significance and variance of each gene’s expression level using Bayesian estimation. Multiple testing corrections were performed using false discovery rate (FDR) correction of *p*-values, and genes with |logFoldchange| > 1 and FDR < 0.05 were selected as differentially expressed genes. GO enrichment analysis was performed using the “clusterProfiler” package (Wu et al., 2021) with FDR correction of *p*-values, the ontology parameter set to Biological Process, and the top 5 terms selected for visualization.

### oposSOM analysis

The oposSOM analysis was performed using the R package “oposSOM” (Löffler-Wirth et al., 2015). The gene expression matrix was passed to the function with the dimension parameter of the first SOM set to 20. This is a package based on Self-Organizing Maps (SOM) that offers a method for extracting inherent functional modules and clusters from data. In short, oposSOM analysis involves projecting a high-dimensional gene expression matrix into a lower-dimensional space of specified dimensions. Within this reduced space, individual units referred to as “meta-genes” emerge, representing genes with closely related expression patterns. By plotting meta-gene expression levels for each sample, we generate meta-gene expression maps. Through the overlap of these sample-specific maps, we can deduce concurrently overexpressed or uniquely expressed meta-genes across various groups. Metagenes overexpressed within specific groups are clustered together and termed as “spots,” representing distinctive characteristic metagenes within specific groups. Subsequently, conducting enrichment analysis on genes within these spots yields valuable insights into gene functionalities. The oposSOM analysis results from oposSOM generated file, enrichment of content including clustering analysis, analysis and specific analysis and visualization of the results. For the detailed calculation procedure of the sub-part, please refer to the literature (Löffler-Wirth et al., 2015).

### Weighted correlation network analysis (WGCNA) and hub gene selection

We performed gene co-expression network analysis using the WGCNA package (Langfelder and Horvath, 2008). According to the user manual, we first performed quality control on genes and samples, selected a soft-thresholding value based on expression data, and constructed a gene co-expression network model. Genes with similar expression patterns were clustered into the same module, and the correlation between modules and clinical phenotypes was calculated. The genes in the MEyellow and MEred modules and the calculated gene connectivity data were extracted and imported into Cytoscape software (Shannon et al., 2003). The MCODE plugin was used to select hub genes.

### Glycolysis score

The glycolysis gene set score was calculated using the ssGSEA method of the “GSVA” package (Hänzelmann et al., 2013), which uses a Gaussian function as the kernel density function to calculate the expression levels of eight glycolysis genes for each sample, resulting in a score.

### Single-cell sequencing analysis

Raw single-cell data was downloaded from the GEO database and processed using the Seurat package (Hao et al., 2021). Quality control was performed based on three indicators: the number of detected genes, the number of unique molecular identifiers, and the percentage of mitochondrial genes. Cells with gene numbers > 200, unique molecular identifiers (UMI) numbers > 500, and mitochondrial gene percentages < 20% were retained. The data were standardized, scaled, and dimensionally reduced according to the reference method in the software package, and the top 12 principal components were selected for cell clustering analysis at a resolution of 0.6. Cell annotations were performed based on reported single-cell markers of placental cells (Liu et al., 2018; Suryawanshi et al., 2018; Pique-Regi et al., 2019; Li et al., 2020).

### Sample collection

The placental samples used in this study were obtained from participants at the First Affiliated Hospital of Chongqing Medical University. Patients were diagnosed according to The American College of Obstetrics and Gynecology’s clinical practice guideline. Patients with known chronic hypertension, gestational complications other than preeclampsia, severe gestational pre-existing conditions, or any co-existing systemic diseases were excluded from the study. Placental tissue was collected using sterile scissors within 5 min of placental delivery during caesarean section and washed in cold PBS solution before fixation with 4% paraformaldehyde and embedding in paraffin. Detailed information regarding placental sample collection can be found in the author’s previous publications (He et al., 2021).

### Histological analysis

The human placental sections were embedded in paraffin and cut to a thickness of 3 μm. Tissue paraffin sections were stained using hematoxylin and eosin (HE) dyes (G1120-3, Solarbio, China). Antigen retrieval was carried out using citrate antigen retrieval buffer (pH 6.0), and endogenous peroxidase was blocked with 3% H2O2 for 25 min. A primary antibody against HK2 (1:200, 66974-1-Ig, Proteintech, China) was utilized for immunohistochemical staining (IHC), and signal detection was performed through diaminobenzidine (DAB) staining using AFIHC004 kit (AIFang Biological, China). The prepared sections were observed and photographed under microscope.

### Cell culture

The HTR8/SVneo cell line used in this study was purchased from the American Type Culture Collection (ATCC, United States). The cells were cultured in recommended Roswell Park Memorial Institute (RPMI) 1,640 medium supplemented with 10% fetal bovine serum (Gibco, United States) and 1% penicillin-streptomycin. The cells were maintained under stable conditions of 37°C, 5% CO2, and 20% O^2^. For hypoxic treatment, the O^2^ concentration was adjusted to 1%, while the other conditions were the same as for normoxic conditions.

### Western blotting

Cell lysates were scraped and dissolved in RIPA buffer (Beyotime, China) containing PMSF (1:100, Beyotime, China) on ice, followed by centrifugation at 12,000 g for 15 min to collect the protein in the supernatant. The protein concentration was standardized and mixed with Laemmli Sample Buffer (#4006028, Bio-Rad, United States) and DTT, followed by separation on a 10% discontinuous SDS-PAGE gel and transferred onto polyvinylidene difluoride membranes (Merck Millipore, GER). The membranes were blocked with 5% skim milk in Tris-buffered saline containing 0.05% Tween-20 for 1 h, and then incubated with specific primary antibodies overnight at 4°C. After that, the membranes were incubated with horseradish peroxidase-conjugated goat anti-mouse IgG or goat anti-rabbit IgG for 1 h at room temperature. Band densitometry was performed using the Quantity One System image analyzer (Bio-Rad, United States). The antibodies used in this study included ATG5 (1:1,000, ab108327, Abcam, United States), BECLIN1 (1:1,000, ab207612, Abcam, United States), LC3 (1:1,000, 14600-1-AP, Proteintech, China), P62 (1:1,000, ab109012, Abcam, United States), and β-Actin (1:1,000, Cell Signaling Technology, United States). All original gel images generated from Western blotting are included in Supplementary Figure S9.

### Statistical analysis and visualization

All statistical analyses in this study were performed using R software (R version 4.2.3) (R Core Team, 2023). In the initial stages of the study, the “pwr” package was utilized for sample size estimation (Stephane, 2020). Based on the research design with four groups, it was estimated that having a sample size greater than 58.67 within each group would provide the study with over 90% power at a medium effect size to detect inter-group differences. In this study, the sample sizes for all groups exceeded this threshold. The entropy value of each sample was calculated using the “entropy” package, with the unit specified as “log2” (Strimmer, 2021). The clustering of LOPE samples was accomplished using the k-means algorithm. Silhouette scores were calculated for various values of the parameter “k” (number of clusters), and the optimal value of “k” was selected. In this study, we employed One-way Analysis of Variance (One-way ANOVA) and two-tailed Student’s t-test for normally distributed and homoscedastic continuous numerical variables to analyze the average differences across multiple and two groups. We employed the Kruskal–Wallis test for multiple groups and the Wilcoxon rank-sum test for two groups to compare average values of non-normally distributed continuous numerical variables. For the statistical methods used in gene differential analysis and enrichment analysis, please refer to the “Differential Analysis and GO Enrichment Analysis” section in the Materials and methods chapter. For numerical correlation analysis, we conducted statistical tests using Pearson correlation analysis. The FDR was used for multiple hypothesis testing. The threshold for type I error was set at *α* = 0.05.

## Results

### Dataset selection, quality control, and pre-processing

**FIGURE 1:**
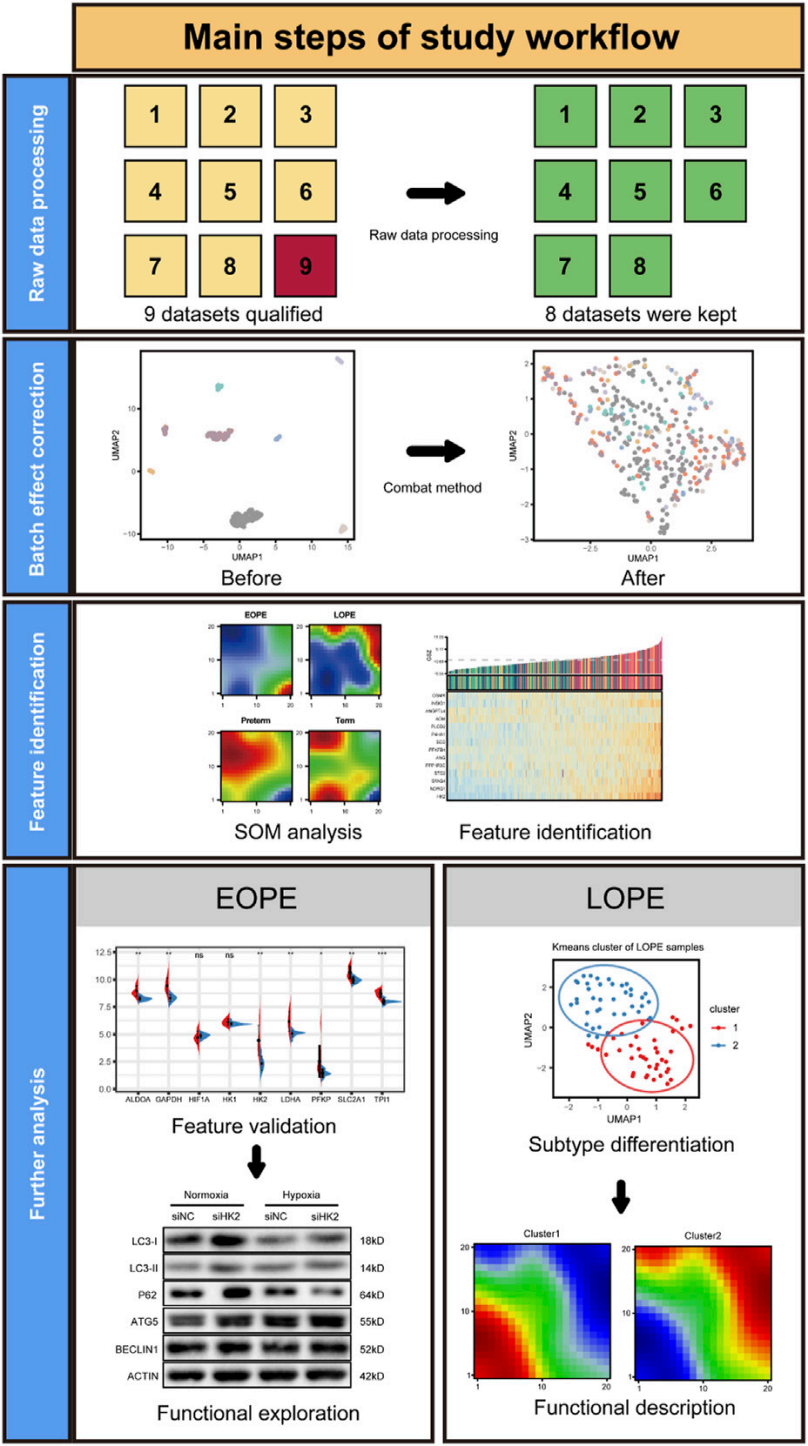
Graphical summary of the study workflow.

The main workflow of this study is illustrated in Figure 1, which includes four steps: raw data processing, batch correction, feature selection and validation, and in-depth analysis. There are a total of nine datasets containing sample information, which meet the criteria for inclusion of similar samples, serving as candidate data. The original data from each dataset undergo quality control and annotation according to the chip design scheme (Supplementary Figure S1). One microarray with significant difference in gene expression distribution was removed (Supplementary Figure S2A). Finally, eight expression matrices were used for integration (Supplementary Figure S2B). There was no significant difference in fetal sex among the groups (Supplementary Table S1).

### Integration of datasets and batch correction

**FIGURE 2:**
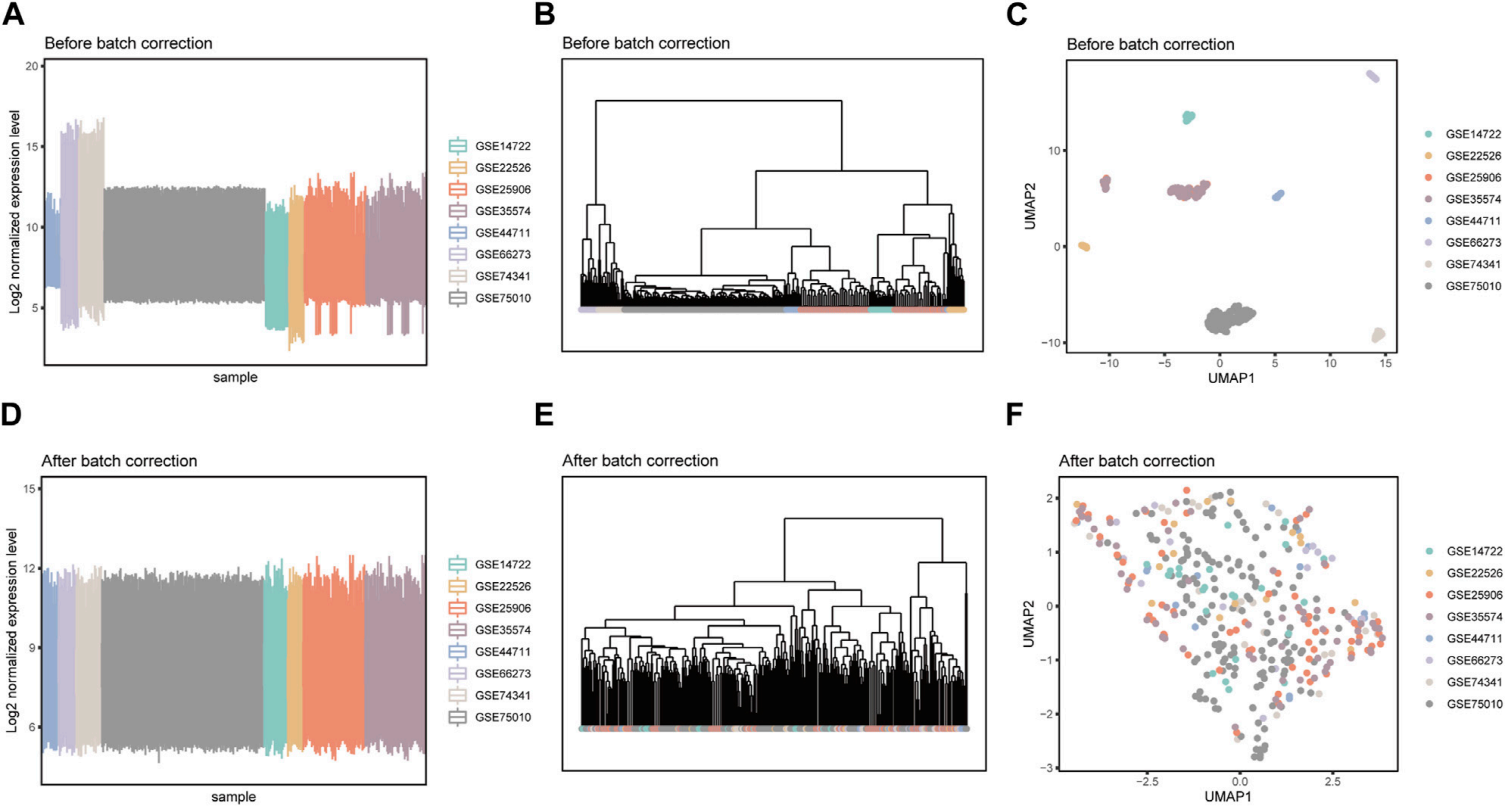
Detection of batch effects before and after data integration. Box plots of sample mean expression levels, hierarchical clustering dendrograms, and umap dimensionality reduction scatter plots before **(A–C)** and after **(D–F)** batch effect correction.

Due to the fact that these datasets are from independently conducted studies, there exists a certain degree of batch effects in the gene expression levels among samples, resulting in a clear batch-dependent distribution in the clustering and dimensionality reduction analyses (Figures 2A–C). After applying the Combat software to address batch effects in the datasets, gene expression levels were normalized, eliminating batch-related distributions (Figures 2D–F). This revealed group-specific distributions instead (Supplementary Figure S3), while retaining the inherent gene expression distribution patterns (Supplementary Figure S4). The integrated matrix after batch correction provides a basis for further analysis (Supplementary Table S2).

### EOPE may represent a homogeneous and independent subtype with typical molecular features of PE placenta

**FIGURE 3:**
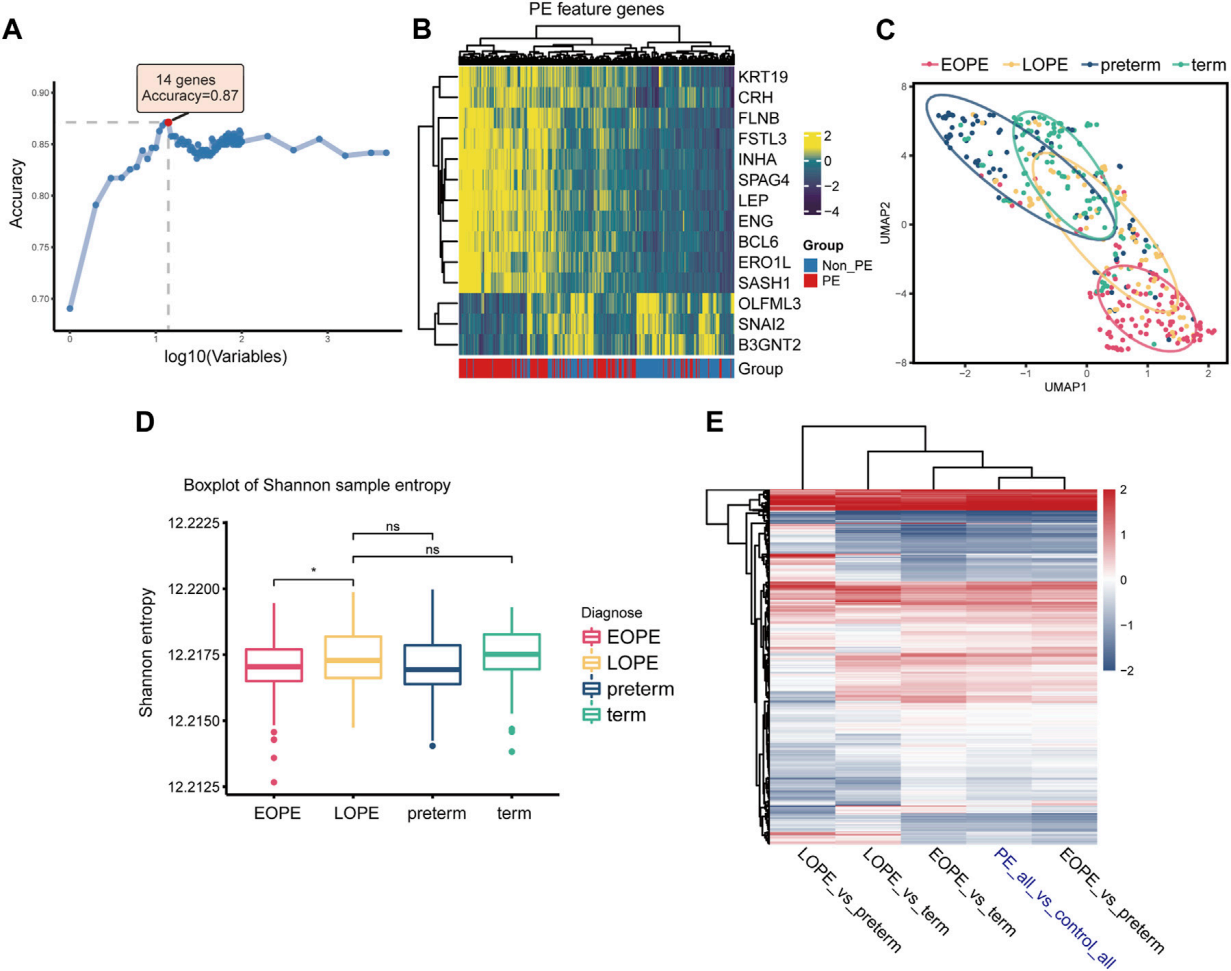
EOPE may represent a homogeneous and independent subtype with typical molecular features of PE placenta. **(A)** Accuracy of random forest models with different numbers of variables in distinguishing between PE and non-PE samples. **(B)** Heatmap depicting the expression levels of PE feature genes in the placenta samples. **(C)** Scatter plot of placenta samples after umap dimensionality reduction on PE feature genes expression, with the elliptical curve representing the 60% confidence interval range. **(D)** Box plots of sample Shannon entropy levels, with the length of the error bars representing the variation of within-group sample differences. Student’s t-test was used for statistical comparison between two groups. “ns” represents no statistical difference, and “*” represents *p* < 0.05. **(E)** Heatmap of differentially expressed genes under different comparison group pairs, with color representing fold change. Here, “PE_all” refers to all preeclampsia samples, including both EOPE and LOPE groups, while “control_all” refers to two types of control samples, encompassing both term and preterm groups.

We first employed RF-RFE, a machine learning algorithm for feature extraction, to screen for PE-related feature genes at different feature set sizes. Results showed that the model containing 14 feature genes had the greatest classification accuracy for grouping (Figure 3A), with 11 upregulated and 3 downregulated in PE (Figure 3B). In terms of these PE feature genes, the EOPE group showed significant homogeneity, while the LOPE group lacked it (Figure 3C). The Shannon entropy of the EOPE samples was also significantly lower than that of the LOPE samples, and the Shannon entropy of LOPE did not differ significantly from that of the two control groups, indicating that the gene expression of the EOPE samples was more deterministic (Figure 3D). The analysis of differential gene expression showed that the fold changes in EOPE were similar to the differences in overall PE samples, whereas LOPE showed significant differences, possibly due to the distinct differential expression patterns between EOPE and LOPE (Figure 3E). To eliminate the influence of the sample size advantage of EOPE, we balanced the sample size by using within-group random sampling for the same analysis and obtained the same conclusion (Supplementary Figure S5). These results suggest that EOPE may be a particularly homogeneous subpopulation in PE.

### Placental transcriptome phenotypes identification reveals higher hypoxia and glycolysis in EOPE but not in LOPE

**FIGURE 4:**
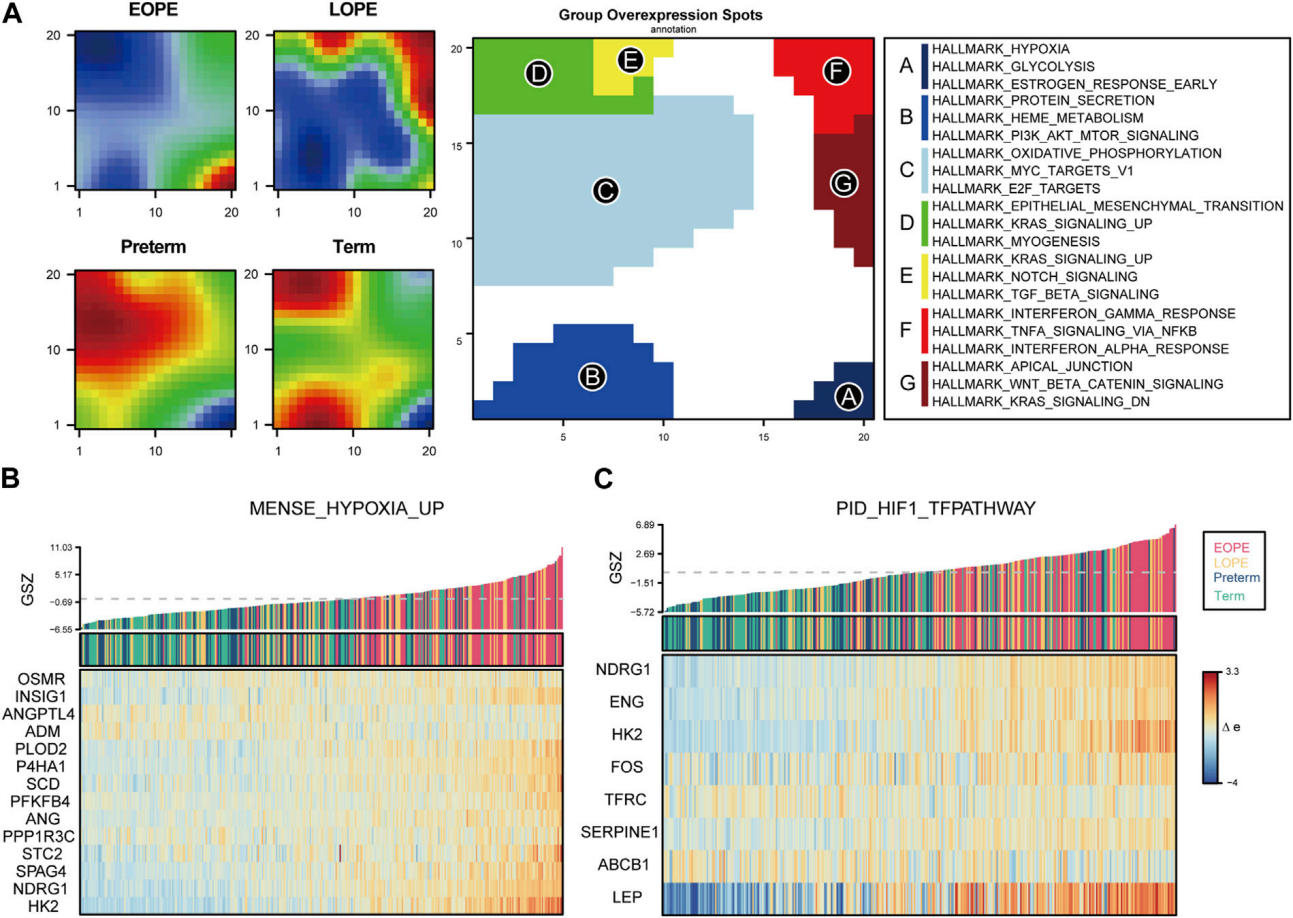
oposSOM analysis indicates that hypoxia is a typical feature of EOPE. **(A)** Matrix of meta-genes and functional annotations obtained by oposSOM clustering. The left four panels represent the expression levels of meta-genes in different groups. Red and blue represent up- and downregulated meta-genes in that group, respectively. The middle panel represents the high-expression meta-genes that compose the spot in the clustering algorithm. The right panel represents the top enrichment results of spot’s HALLMARK gene set. **(B–C)** Bar plots of Gene Set Z-scores of the MENSE_HYPOXIA_UP and PID_HIF1_TFPATHWAY gene sets and heatmap of their gene expression levels in all samples.

**FIGURE 5:**
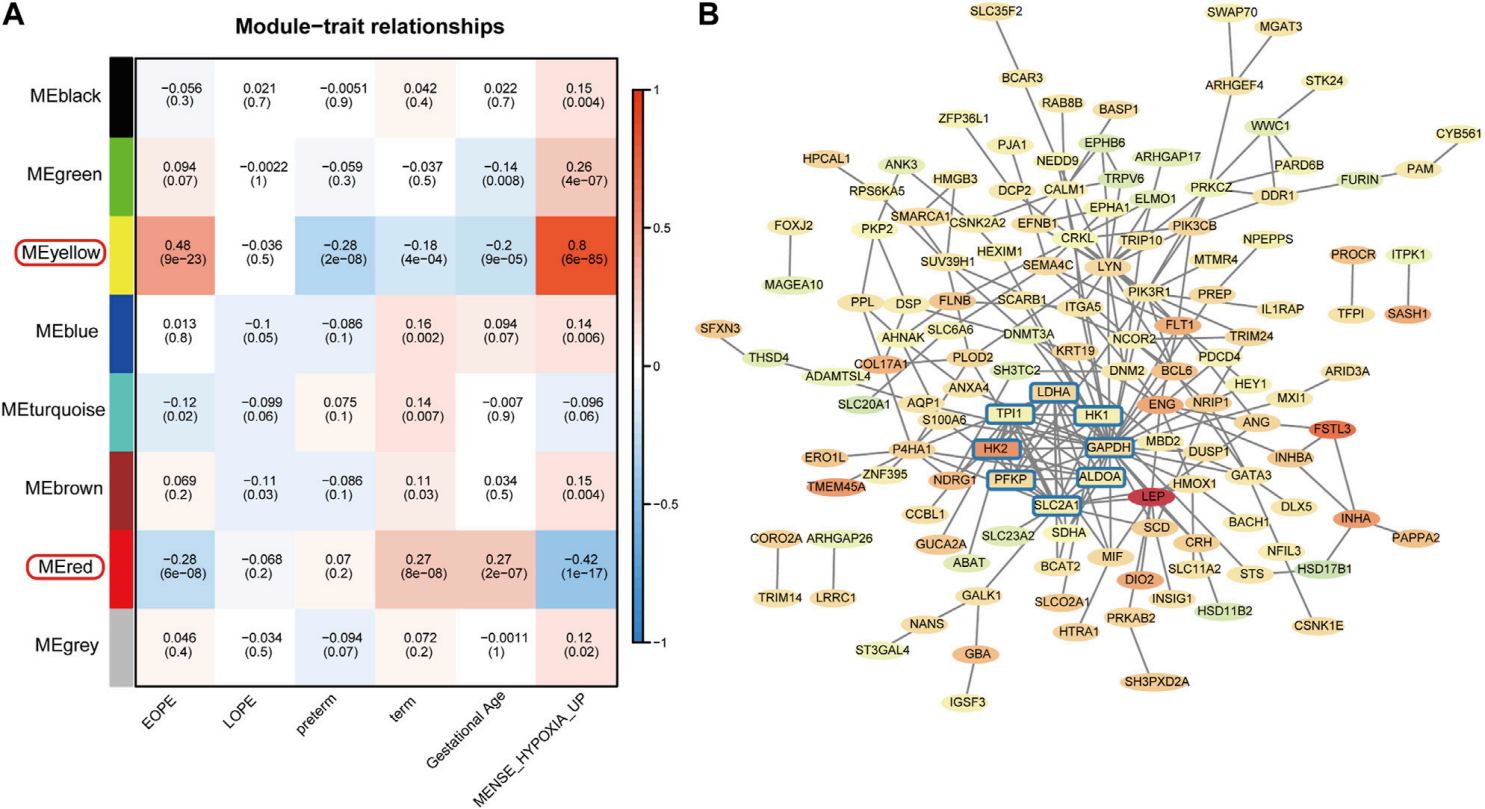
Filtering of EOPE phenotype-associated genes and analysis of highly connected genes. **(A)** Heatmap of the correlation between gene modules and sample phenotypes. The first row of numbers in each cell represents the Pearson correlation coefficient R, and the numbers in parentheses in the second row represent the *p*-value. The yellow and red modules, which are most correlated with EOPE, were selected for the filtration of core genes. **(B)** Selection of EOPE-correlated genes and hub genes. The red or green background represents genes that are upregulated or downregulated in the EOPE group, with the term group serving as the control. The color intensity reflects the magnitude of the difference. The rounded square genes within the blue box represent hub genes with the highest connectivity among EOPE-correlated genes.

To explore the homogeneity and heterogeneity of different subtypes, we employed an unsupervised neural network-based self-organizing map method. Genes with similar expression patterns were merged into meta-genes (Supplementary Table S3), and meta-genes within specific group, called group overexpression spots, were analyzed for functional enrichment (Supplementary Table S4). Consistent with the known features of EOPE placenta, EOPE displayed a phenotype characterized by hypoxia and glycolysis, which was absent in both control groups, while LOPE only partially exhibited this phenotype (Figure 4A). LOPE enriched the least in specific gene sets, suggesting its features were less distinct (Supplementary Table S5). In addition, gene-level analysis showed that hypoxia-related genes were significantly upregulated in EOPE (Figures 4B,C). These results suggest that the main feature of EOPE is the significant upregulation of hypoxia-related genes, while the features of LOPE are relatively less apparent. To identify the core genes regulated by hypoxia and HIF1, we used WGCNA to find the yellow and red gene modules highly positively and negatively correlated with hypoxia index, respectively (Figure 5A). These two modules also showed the strongest correlation with EOPE phenotype, consistent with our oposSOM results (Figure 4 and Figure 5A]. By calculating the connectivity of genes within the two modules, we obtained the top 8 highly connected genes, including SLC2A1, HK1, HK2, PFKP, ALDOA, TPI1, GAPDH, and LDH (Figure 5B).

### Validation of glycolysis genes and its association with clinical features

**FIGURE 6:**
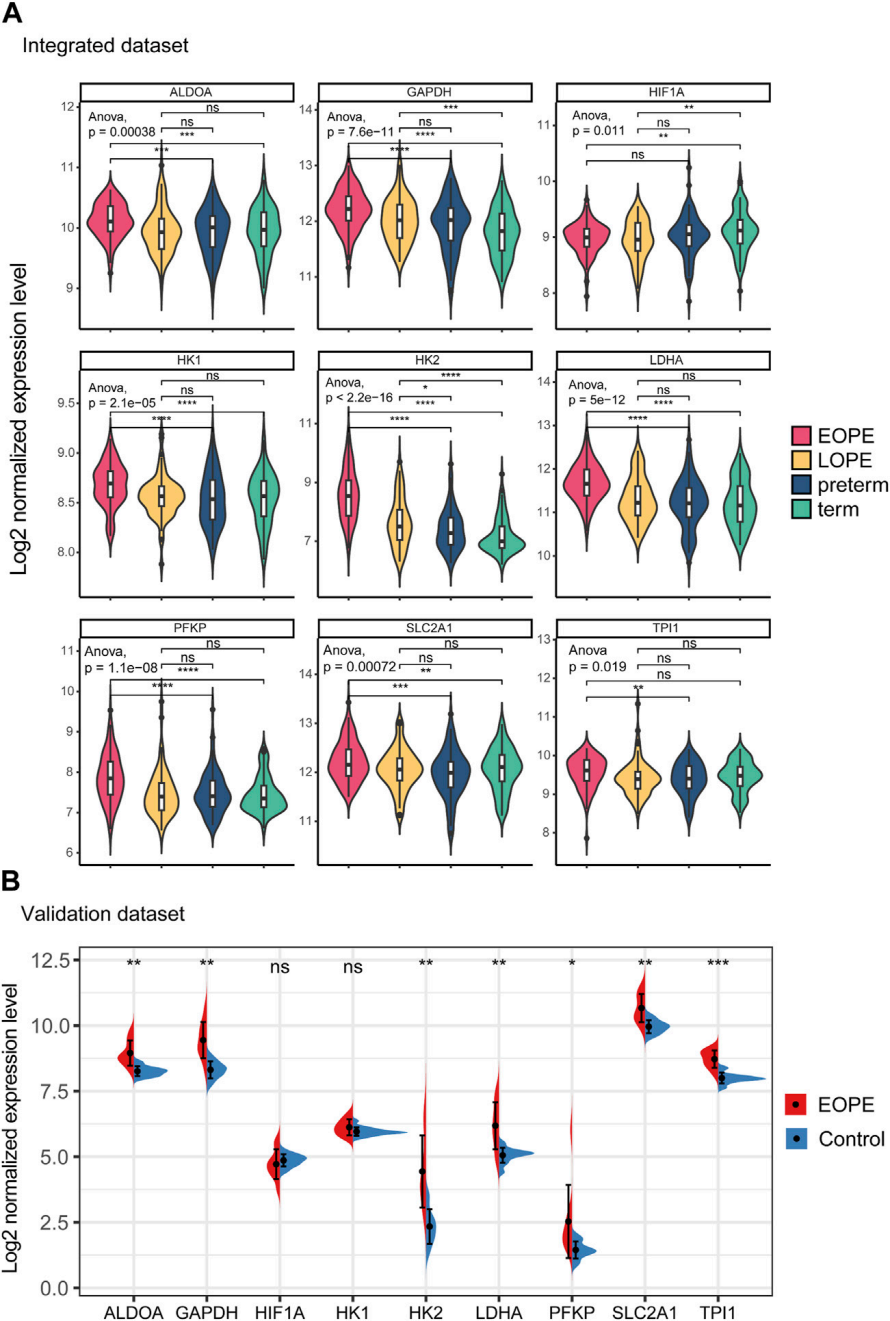
Integration matrix showing upregulated expression of glycolytic genes in EOPE samples and validation of the results in independent datasets. **(A)** Violin plot of the expression levels of glycolysis genes and HIF1A in the integrated dataset samples. One-way ANOVA was used to compare differences between multiple groups, and a Student’s t-test was used for pairwise comparisons. **(B)** Violin plot of the expression levels of glycolysis genes and HIF1A in the validation dataset samples. A Student’s t-test was used to compare differences between groups, with “ns” indicating no statistical difference, “*” indicating *p* < 0.05, “**” indicating *p* < 0.01, “***” indicating *p* < 0.001.

**FIGURE 7:**
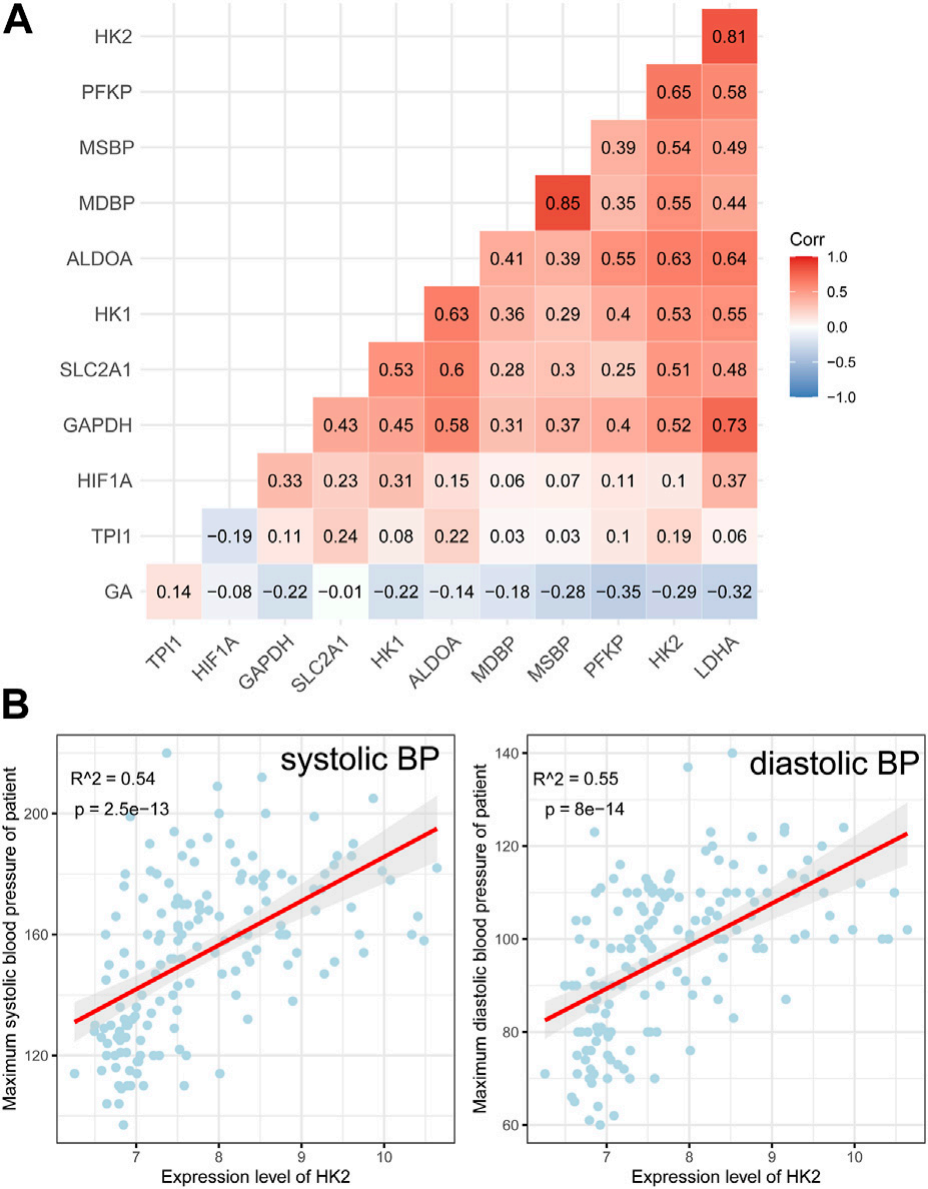
Correlation analysis between glycolysis genes, HIF1A, and clinical features. **(A)** Heatmap showing the correlation between glycolysis genes, HIF1A, maximum systolic blood pressure (MSBP), maximum diastolic blood pressure (MDBP), and gestational age (GA). The numbers within the cells represent the R-values from Pearson correlation analysis, while the intensity of cell color represents the relative magnitude of the R-values; **(B)** Scatter plots and Pearson correlation analysis between HK2 expression and MSBP/MDBP, with a red line indicating the fitted regression line and gray area indicating the 95% confidence interval.

All of these genes were significantly upregulated in the EOPE group and were key genes in the glycolytic pathway, but were less upregulated in LOPE (Figure 6A). Compared with the term group, the transcription level of HIF1A was lower in both PE subtypes (Figure 6A). In another independent data set based on sequencing technology, the upregulation of these eight genes in EOPE placenta was validated, except for HK1 (Figure 6B), while HIF1A showed no significant difference in validation (Figure 6B). Besides the gene level, GSEA analysis also showed that multiple pathways related to glycolysis were enriched in EOPE and were validated in the validation set (Supplementary Figure S6). EOPE had a higher glycolysis score compared to the control group in validation dataset (Supplementary Figure S7A). When using these eight glycolysis genes for dimensionality reduction analysis of the integration matrix, a distribution result similar to the PE feature gene dimensionality reduction was obtained, which could clearly distinguish EOPE samples (Supplementary Figure S7B). In addition, HK2 had the highest correlation with blood pressure, but was almost uncorrelated with gestational age (Figure 7). Therefore, we believe that the glycolysis pathway may represent a distinctive feature of EOPE that is absent in LOPE.

### Single-cell analysis reveals upregulation of glycolysis in EOPE trophoblast cells

**FIGURE 8:**
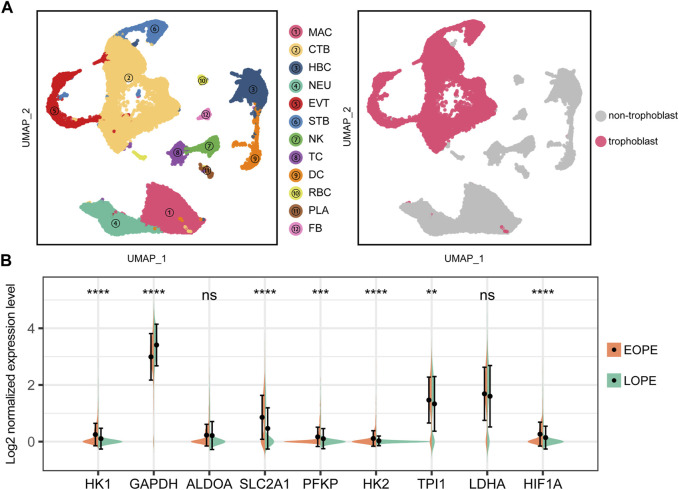
Single-cell analysis suggests upregulation of glycolysis genes and HIF1A in EOPE trophoblasts compared to LOPE. **(A)** Left panel: UMAP dimensional reduction scatter plot and cell type annotation of placental cells. (2PE placenta samples vs. 2conrtol placenta samples) at the single-cell level. Right panel: Isolation of trophoblast cells for further analysis. MAC: macrophage; CTB: cytotrophoblast; HBC: hofbauer cell; NEU: neutrophil; EVT: extra-villous trophoblast; STB: syncytiotrophoblast; NK: natural killer cell; TC: T cell; DC: dendritic cells; RBC: red blood cell; PLA: platelets; FB: fibroblast; **(B)** Violin plot of the expression levels of glycolysis genes and HIF1A in trophoblast cells from EOPE and LOPE samples. A Student’s t-test was used to compare differences between groups, with “ns” indicating no statistical difference, “*” indicating *p* < 0.05, “**” indicating *p* < 0.01, “***” indicating *p* < 0.001, and “****” indicating *p* < 0.0001.

The trophoblast, as a central and crucial type of cells composing the placenta, was the focus of our investigation on the possible aberrant upregulation of the glycolytic pathway. To this end, we analyzed a publicly available single cell dataset comprising samples of both EOPE and LOPE after rigorous quality control and annotation procedures (Supplementary Figure S8). Subsequently, we extracted the trophoblast cells for further investigation (Figure 8A). Our findings indicated that the glycolytic genes were also upregulated in EOPE trophoblast cells, except for GAPDH and LDHA (Figure 8B). Moreover, the transcriptional level of HIF1A was found to be elevated in the EOPE trophoblast cells (Figure 8B). These results suggest that the upregulation of glycolysis is indeed a prominent characteristic of EOPE, which is present in trophoblast cells.

### Possible association between HK2 elevation in EOPE placental trophoblast and regulation of autophagy

**FIGURE 9:**
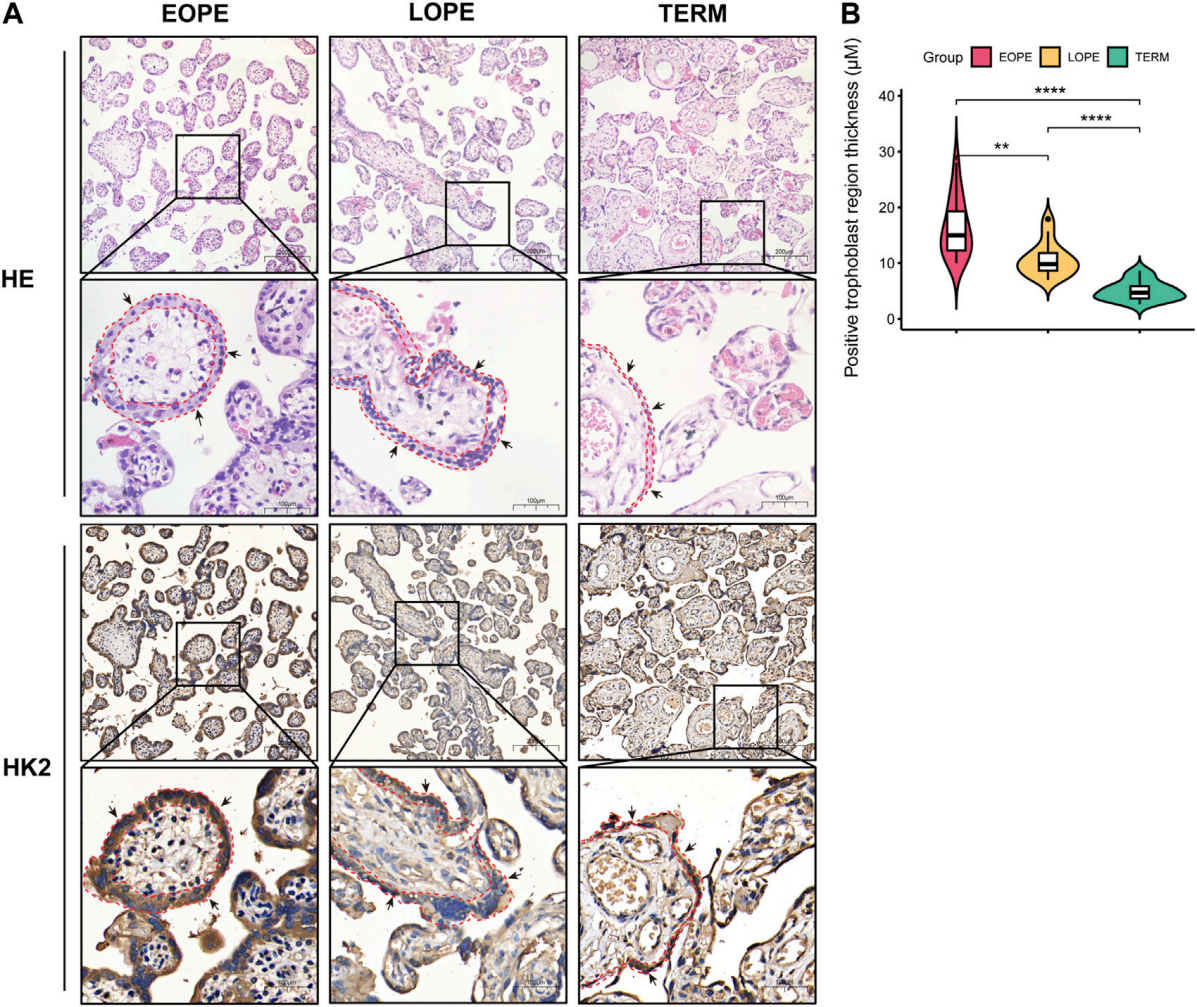
Histological analysis of placental samples from EOPE, LOPE, and term pregnant women. **(A)** Histological staining of tissue sections with HE and IHC for HK2. For both HE and IHC results, the magnification of the upper cells is ×20, with a scale bar length of 200 μm. The magnification of the lower cells is ×40, with a scale bar length of 100 μm. The black arrows indicate syncytiotrophoblast, while the red dashed lines delineate the area containing syncytiotrophoblast in the section. Each group was analyzed using one sample. **(B)** Violin plots of the thickness of the HK2-positive trophoblast layer determined by measuring 15 randomly selected villi region in each IHC staining slide. The statistical significance of the difference between the two groups was determined using a Student’s t-test. Each group had one slide, with no biological replicates.

**FIGURE 10:**
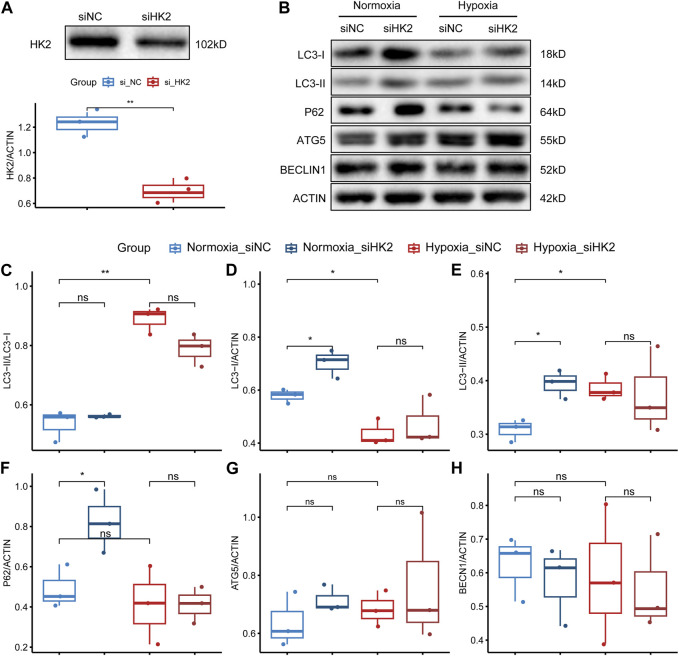
HK2 may regulate the expression of autophagy-related markers in trophoblasts. **(A)** Western blotting and statistical analysis of HK2 protein expression after siRNA interference of HTR8/SVneo. The upper panel shows the Western blotting result of HK2, and the lower panel shows the statistical graph of the band density of HK2. The biological replicates were three, and the Student’s t-test was used to test the difference between groups. **(B)** Western blotting results of the expression levels of LC3, P62, ATG5, and BECLIN1 in siHK2-interfered HTR8/SVneo cells under normoxic and hypoxic conditions. **(C–H)** Statistical graphs of the band density values of the Western blotting results with three biological replicates. The Student’s t-test was used to compare the differences between the two groups. Statistical significance marks: “ns” indicating no statistical difference, “*” indicating *p* < 0.05, “**” indicating *p* < 0.01, “***” indicating *p* < 0.001, and “****” indicating *p* < 0.0001.

Based on our analysis, HK2 was found to be the gene with the highest upregulation among these glycolysis-related genes, and showed the strongest correlation with both systolic and diastolic blood pressure (Figures 6–8). Thus, we performed HE staining and IHC staining of HK2 in tissue, which showed that HK2 is localized in the syncytiotrophoblast layer of the placenta, and that EOPE samples had a thicker syncytiotrophoblast layer compared to control samples (Figure 9). Given the previously reported tight connection between HK2 and autophagy, as well as the known relationship between autophagy and PE, we sought to investigate whether HK2 might regulate autophagy in trophoblast cells. We therefore inhibited HK2 expression in HTR8 cells (Figures 10A,B) under normoxic and hypoxic conditions, and assessed the expression of autophagy markers. Hypoxia increased the ratio of LC3-II/LC3-I and decreased the level of LC3-I in HTR8 cells, indicating an induction of autophagy in trophoblast cells under hypoxia (Figures 10C,D). Under normoxic conditions, the decrease in HK2 expression caused a significant increase in LC3-I and P62 levels, suggesting that HK2 may have an inhibitory effect on autophagy (Figures 10C,F). In fact, the level of LC3-II also increased, but to a lesser extent than LC3-I (Figures 10D,E). However, under hypoxia conditions, HK2 had no effect on the expression levels of LC3 and P62 (Figures 10C–F). Additionally, both hypoxia and HK2 levels did not appear to have a significant effect on the levels of ATG5 and BECLIN1 (Figures 10G,H). These results suggest that HK2 may inhibit autophagy in trophoblast cells under normoxic conditions, but not under hypoxic conditions.

### Clustering of LOPE placental samples reveals two groups with distinct functional features

**FIGURE 11:**
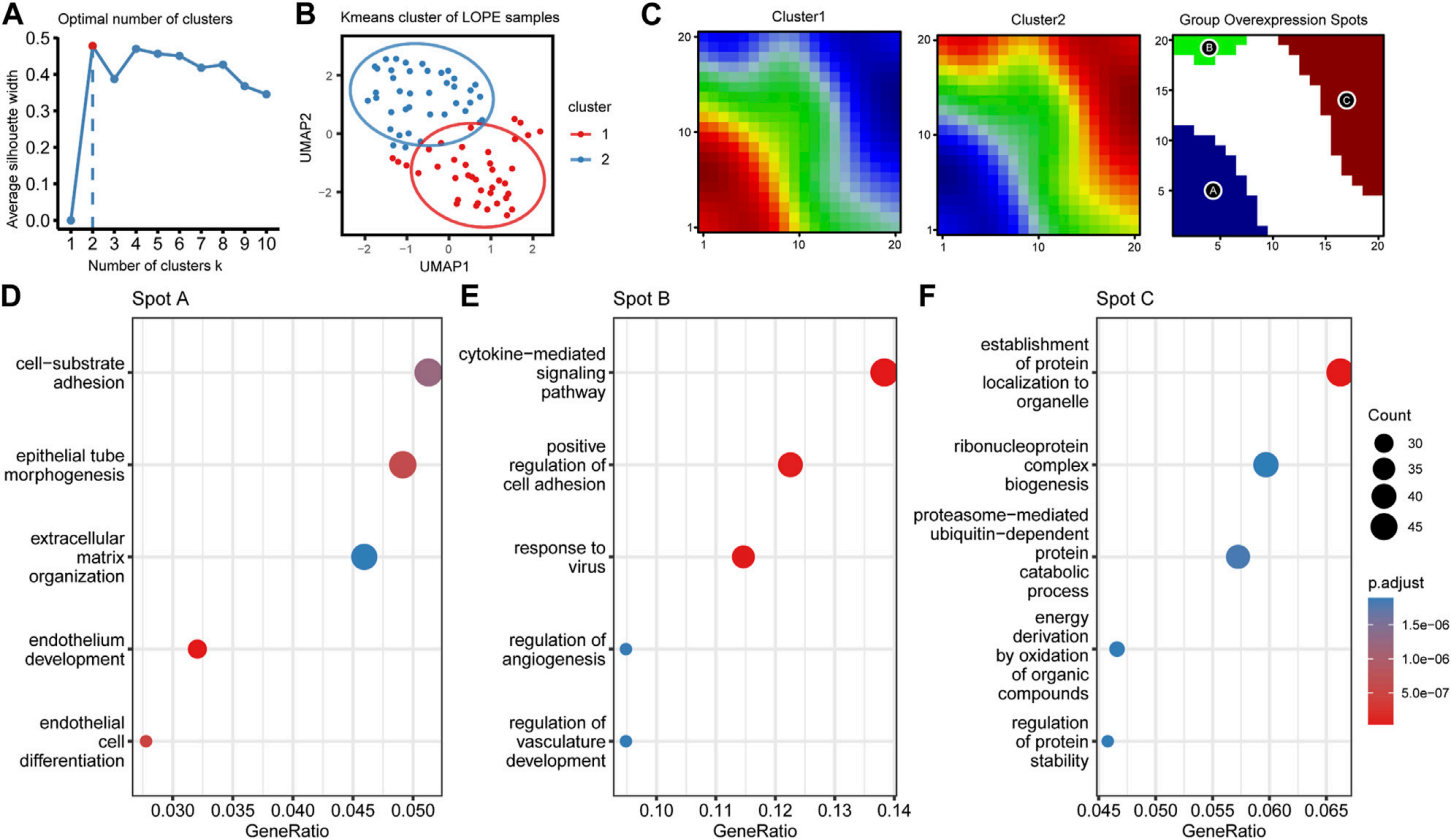
LOPE samples can be divided into two subgroups with different functional characteristics using unsupervised clustering. **(A)** The average silhouette width of LOPE samples under different k values in Kmeans unsupervised clustering. **(B)** The scatter plot of LOPE samples after umap dimensionality reduction when k = 2, with the ellipse circle representing the range of 90% confidence interval. **(C)** Metagene matrix and group overexpression spots of oposSOM analysis for the two LOPE sample groups. **(D–F)** GO enrichment analysis results for the top spot A-C.

At last, due to significant heterogeneity in the LOPE placental transcriptome, we performed clustering analysis on LOPE samples to determine whether any potential subtypes exist. K-means algorithm was employed for sample clustering, and when clustering into two classes, the clusters exhibited the greatest differentiation between them (Figures 11A,B). The oposSOM algorithm was then used to extract characteristic genes of the two clusters (Supplementary Table S6), where cluster 1 was characterized by spot A, while cluster 2 was characterized by spots B and C (Figure 11C). Enrichment analysis was conducted on the genes of the three spots to obtain functional annotations of the spot features. Spot A genes were found to primarily regulate epithelial formation and matrix composition (Figure 11D), Spot B genes regulate cytokines and vascular generation (Figure 11E), while Spot C genes regulate protein production, transposition, and degradation (Figure 11F).

## Discussion

Normal development and proper functioning of the placenta are essential prerequisites for a healthy pregnancy (Ortega et al., 2022). Abnormalities in the placenta can lead to damage in multiple systems, including the heart, brain, and vasculature (Ortega et al., 2023a; 2023b). Among these, vascular damage is a primary mechanism underlying the onset of PE. Accumulating evidence suggests that EOPE and LOPE are the two most meaningful subtypes (Jung et al., 2022; Magee et al., 2022), however, the characteristics of these two subtypes are largely unknown. In this study, we found that EOPE had stronger sample homogeneity and significant differences from preterm and term samples, while LOPE samples had no apparent features. This result is consistent with the results of two other studies that showed that the number of differentially expressed genes in EOPE compared to the control group is much greater than in LOPE (Liang et al., 2016; Guo et al., 2021). The results suggest significant pathological changes in EOPE placenta and minor changes in LOPE placenta. In addition, another study including 1,691 placental samples grouped the samples based on clinical symptoms and histopathology, reporting a unique subtype of MVM with poor maternal vascularization, which has more severe symptoms and smaller fetuses (Horii et al., 2023). Encouragingly, this subgroup has the smallest gestational weeks among all PE samples (Horii et al., 2023). The results imply that more severe symptoms and smaller fetuses may be associated with earlier gestational weeks. Additionally, the significant changes in placental gene expression indicate that EOPE is a highly specific subtype of PE. Based on this finding, it may be advisable to consider recognizing and managing EOPE as a distinct clinical entity.

Although these studies have recognized the unique status of EOPE in PE, there have been some discrepancies among different studies regarding the specific molecular pathological features of EOPE. These studies have identified different differentially expressed genes and summarized different features of EOPE, including changes in G protein-coupled receptors, angiogenesis, and innate immunity (Junus et al., 2012; Liang et al., 2016; Broekhuizen et al., 2021). While these results may coexist, they are also confusing. Limited sample size and varied control groups may contribute to discrepancies in the detection of different aspects of EOPE features across studies. We resolved this issue by integrating extensive placental data and incorporating two commonly used control samples, thereby mitigating any potential bias resulting from varying control groups. Our findings indicate that the activation of hypoxia and HIF1 signaling is the primary characteristic of EOPE placenta, accompanied by significant alterations in metabolic pathways. While hypoxia can bring about multifaceted changes in cells, our study suggests that the dysregulated activation of glycolysis may be the core response to hypoxia.

Glycolysis and induction of hypoxia genes are manifestations of tissue hypoxia (Bacigalupa and Rathmell, 2020; Kierans and Taylor, 2021). For trophoblasts, appropriate hypoxia in early pregnancy promotes remodeling of the uterine spiral arteries and increases blood flow, preventing placental and fetal ischemia in the mid-to-late stages of pregnancy, which is a necessary process for successful pregnancy (Turco and Moffett, 2019; García-Montero et al., 2022). Obviously, this hypoxia process erroneously persists in EOPE placenta until the mid-to-late stage (Cheng et al., 2022). A recent study utilizing mass spectrometry technology demonstrated elevated lactate levels and decreased Fructose 6-phosphate and Glucose 6-phosphate levels in EOPE placenta, which strongly supports our findings (Kawasaki et al., 2019). Over-activation of glycolysis may cause accumulation of the final product lactate and a decrease in intermediate products, which can be lethal to cells (Hsiao et al., 2009; Li et al., 2022). However, as hypoxia itself can strongly induce the physiological process of glycolysis (Kierans and Taylor, 2021), we speculate that the glycolytic phenotype in EOPE placenta may simply be an adaptive compensation to poor spiral artery remodeling and hypoxia.

Although the main function of these glycolytic genes is to regulate cells’ anaerobic glucose metabolism, they are also often reported to regulate multiple cellular functions (Yun et al., 2015; Gao et al., 2021; Yao et al., 2023). The classical function of the HK2 protein is to act as the first rate-limiting enzyme in the glycolytic pathway, converting glucose entering the cell to glucose-6-phosphate and possessing a strong affinity for glucose (Tan and Miyamoto, 2015; Yuan et al., 2022; Zhao et al., 2022). However, HK2 is also a key gene in integrating glycolysis and autophagy in cells, and its binding to mTORC1 controls the balance between glycolysis and autophagy (Tan and Miyamoto, 2015; Jiao et al., 2018). Our results suggest that HK2 regulates basal autophagic flux under normoxic conditions by inhibiting cell autophagy, as evidenced by the increase in LC3-II and LC3-I levels and the accumulation of P62 upon reduction of HK2. However, HK2 lost its regulatory effect on these markers under hypoxic conditions. Recent studies have indicated autophagy plays a crucial role in physiological hypoxia-induced vascular remodeling and the invasion of extra-villous trophoblasts (EVTs), a critical subtype responsible for spiral artery remodeling (Nakashima et al., 2017). From these findings, it appears that HK2 is a strong contender as the core gene that underlies the relationship between early pregnancy vascular remodeling, EVT autophagy, glycolysis and changes in oxygen tension.

Besides EOPE, LOPE is another significant subgroup of preeclampsia, accounting for about two-thirds of PE incidence, however, it is less well-understood in medical research (Magee et al., 2022). Our study suggests that accurately characterizing the features of LOPE is challenging due to the potential presence of multiple subgroups with distinct characteristics. Unsupervised clustering analysis indicates that LOPE may have at least two sub-groups. Cluster 1 is characterized by features related to cell-environment interaction, while cluster 2 is associated with cytokine and protein modification. Both clusters seem to emphasize changes in the microenvironment of trophoblasts rather than changes in the larger population of trophoblasts, including cell-matrix interaction and cell-cell interaction. This may be one possible reason why anomalies in LOPE placenta are less apparent than those in EOPE. For example, cluster 1, which is enriched in extracellular matrix and endothelial cell-related functions, may reflect changes in the placental matrix and endothelial cells that primarily exist in cluster 1. Cluster 2, characterized by cytokines, angiogenesis, and protein modification, may also be an expression of the placenta’s response to the microenvironment. Recent reviews suggest that the occurrence of LOPE is associated with systemic inflammation and abnormalities in maternal endothelial regulation secondary to increased fetal placental demand, which appears to be consistent with the characteristics of the two LOPE clusters we identified (Magee et al., 2022). Additionally, studies have reported a correlation between protein modification and the onset of PE (Buhimschi et al., 2014). These findings highlight the distinctions between the two subtypes of PE and imply the existence of at least two clusters within LOPE.

Although we have included an ample number of samples and conducted extensive analyses, certain limitations remain. As a result of integrating gene expression matrices from eight independent studies, certain gene probes have been excluded, leading to coverage of a majority rather than the entire gene information. Additionally, due to the independent origins of the datasets, the absence of consistent and comprehensive clinical feature records makes it challenging to thoroughly assess potential influences of factors such as maternal age and ethnicity on placental molecular expression. Nonetheless, these limitations represent compromises with the current state of available datasets. With the growing prevalence of high-throughput technologies, future datasets are likely to address these issues more comprehensively.

In conclusion, preeclampsia is a multifaceted disease that may have various underlying causes leading to comparable symptoms. Our study suggests that the preeclampsia population may consist of several subtypes with distinct features, highlighting significant discrepancies between EOPE and LOPE and strongly advocating for the independent investigation of these two subtypes. Moreover, HK2 may play a pivotal role in connecting the glycolysis and autophagy of the EOPE placenta. However, our study has limitations since transcriptome analysis commonly faces the challenge of explicating causal relationships between observed phenomena. Therefore, further experiments are necessary to determine whether the upregulation of HK2 is the primary cause of EOPE. Overall, our study may enhance comprehension of the intricate pathogenesis of preeclampsia and provide valuable insights for future research.

## Data Availability

The datasets presented in this study can be found in online repositories. The names of the repository/repositories and accession number(s) can be found in the article/Supplementary Material.

## Abbreviations

PE, Preeclampsia; EOPE, early-onset Preeclampsia; LOPE, late-onset PE Preeclampsia; GEO, Gene Expression Omnibus; HE, hematoxylin and eosin; IHC, immunohistochemical staining; RFE, recursive feature elimination; RF-RFE, random forest recursive feature elimination; FDR, false discovery rate; UMI, unique molecular identifiers; WGCNA, Weighted correlation network analysis; EVT, extra-villous trophoblast.
