# Supplementary material for: Elevated expression of glycolytic genes as a prominent feature of early-onset preeclampsia: insights from integrative transcriptomic analysis
Source: Front Mol Biosci. 2023 Sep 25;10:1248771. doi: 10.3389/fmolb.2023.1248771 (PMC10561389; doi:10.3389/fmolb.2023.1248771)
Supplement: Supplementary file 1 [file DataSheet1.ZIP › supplement tables/supp_table_1.docx]

**Supplement Table 1.** Clinical information of the sample in integration dataset.

|  | **Preeclampsia** | | **Control** | |  |  |  |
| --- | --- | --- | --- | --- | --- | --- | --- |
|  | **EOPE (N=105)** | **LOPE (N=79)** | **preterm (N=83)** | **term (N=105)** | **P.method** | **P.value** | **normality** |
| **Gestational age (weeks)** |  |  |  |  | Kruskal | <0.001 | non-normal |
| Mean (CV%) | 29.9 (8.2%) | 36.3 (4.6%) | 31.1 (11.5%) | 38.3 (2.5%) |  |  |  |
| Missing | 7 (6.7%) | 8 (10.1%) | 5 (6.0%) | 5 (4.8%) |  |  |  |
| **Fetal gender** |  |  |  |  | Chi-square | 0.253 | - |
| Female | 42 (40.0%) | 27 (34.2%) | 25 (30.1%) | 45 (42.9%) |  |  |  |
| Male | 31 (29.5%) | 34 (43.0%) | 34 (41.0%) | 55 (52.4%) |  |  |  |
| Missing | 32 (30.5%) | 18 (22.8%) | 24 (28.9%) | 5 (4.8%) |  |  |  |
| **Delivery mode** |  |  |  |  | Chi-square | <0.001 | - |
| C-Section | 47 (44.8%) | 17 (21.5%) | 28 (33.7%) | 22 (21.0%) |  |  |  |
| Induced | 12 (11.4%) | 18 (22.8%) | 0 (0%) | 15 (14.3%) |  |  |  |
| Spontaneous | 2 (1.9%) | 10 (12.7%) | 11 (13.3%) | 51 (48.6%) |  |  |  |
| Vaginal | 2 (1.9%) | 14 (17.7%) | 15 (18.1%) | 12 (11.4%) |  |  |  |
| Missing | 42 (40.0%) | 20 (25.3%) | 29 (34.9%) | 5 (4.8%) |  |  |  |
