# Supplementary material for: Elevated expression of glycolytic genes as a prominent feature of early-onset preeclampsia: insights from integrative transcriptomic analysis
Source: Front Mol Biosci. 2023 Sep 25;10:1248771. doi: 10.3389/fmolb.2023.1248771 (PMC10561389; doi:10.3389/fmolb.2023.1248771)
Supplement: Supplementary file 2 [file DataSheet2.ZIP › supplement figures.docx]

Supplementary **Figure1**


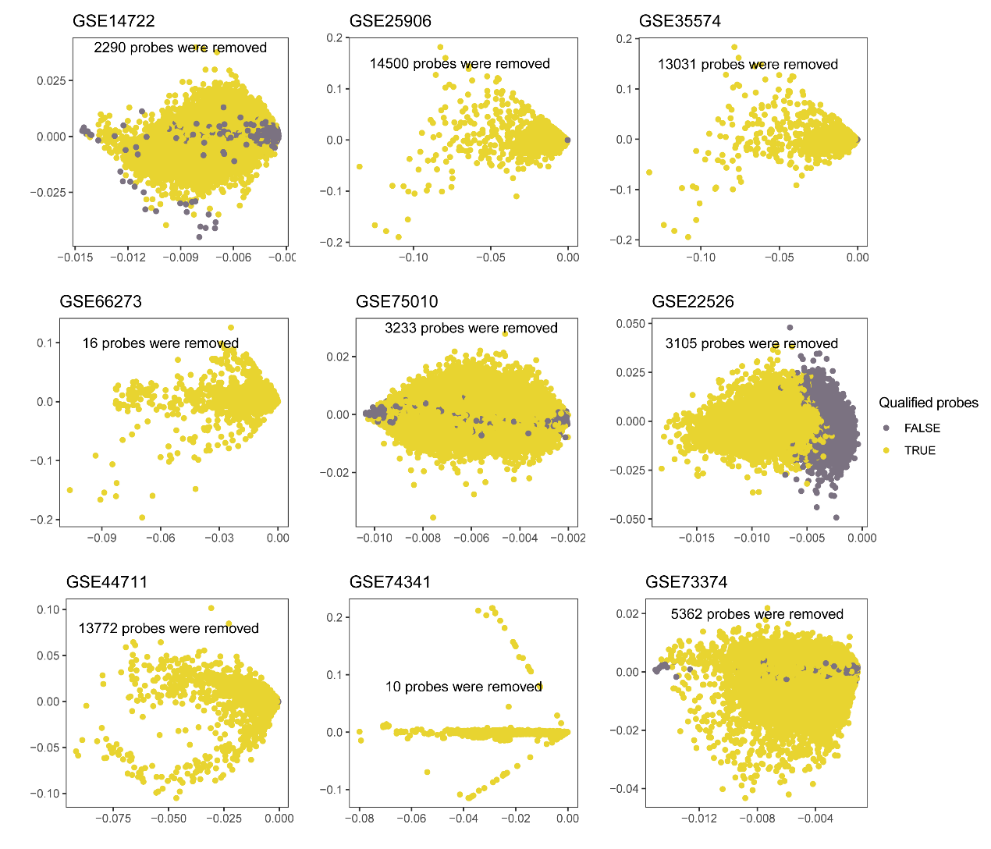


Supplementary Figure 1. PCA plot of probe quality control for candidate datasets. Probes from the dataset were reduced to 2 principal components using PCA. The yellow dots represent probes that meet the quality control standards, while the gray dots represent low-quality probes that were excluded.

**Supplementary Figure2**


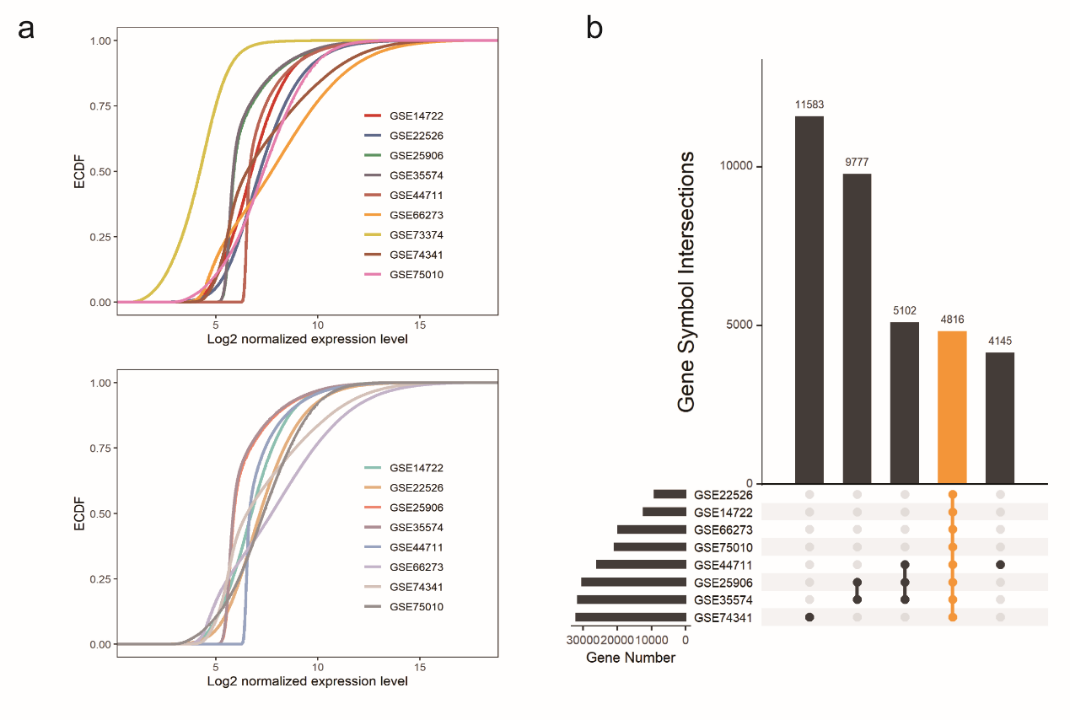


Supplementary Figure 2. Eight candidates from the matrix were ultimately selected for integrated analysis. (a) The upper panel shows ECDF curves of 9 candidate datasets, and the lower panel shows the ECDF curves after removing dataset GSE73374. (b) The UpSet plot shows the number of intersecting genes among the final 8 included datasets.

**Supplementary Figure3**


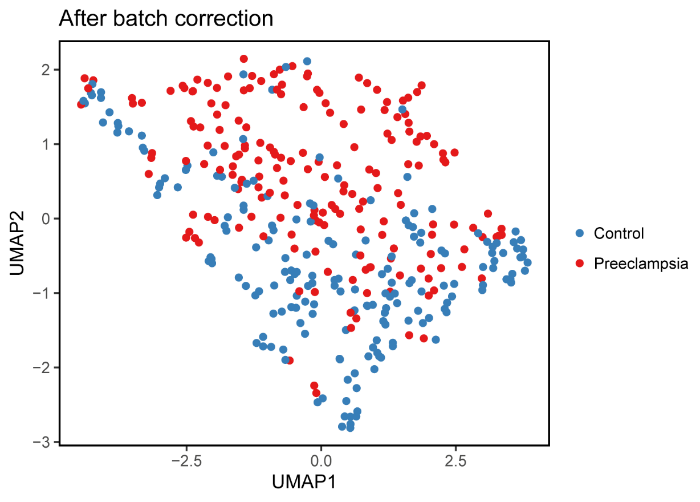


Supplementary Figure 3. Samples after batch effect correction exhibit varying distributions based on different groupings. UMAP dimension reduction scatter plot of the integrated matrix samples after batch effect removal.

**Supplementary Figure4**


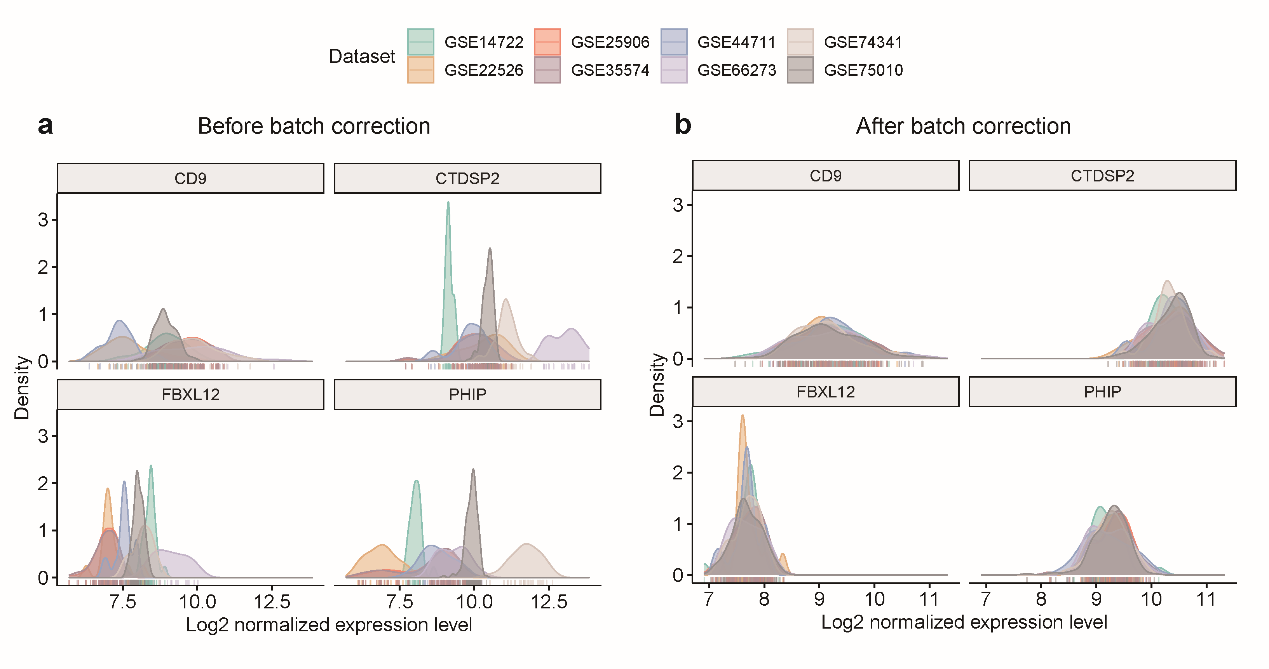


Supplementary Figure 4. The distribution disparities of genes across different datasets are rectified after batch effect correction. Density distribution plots of four randomly selected genes, CD9, CTDSP2, FBXL12, and PHIP before (a) and after (b) batch effect removal.

**Supplementary Figure5**


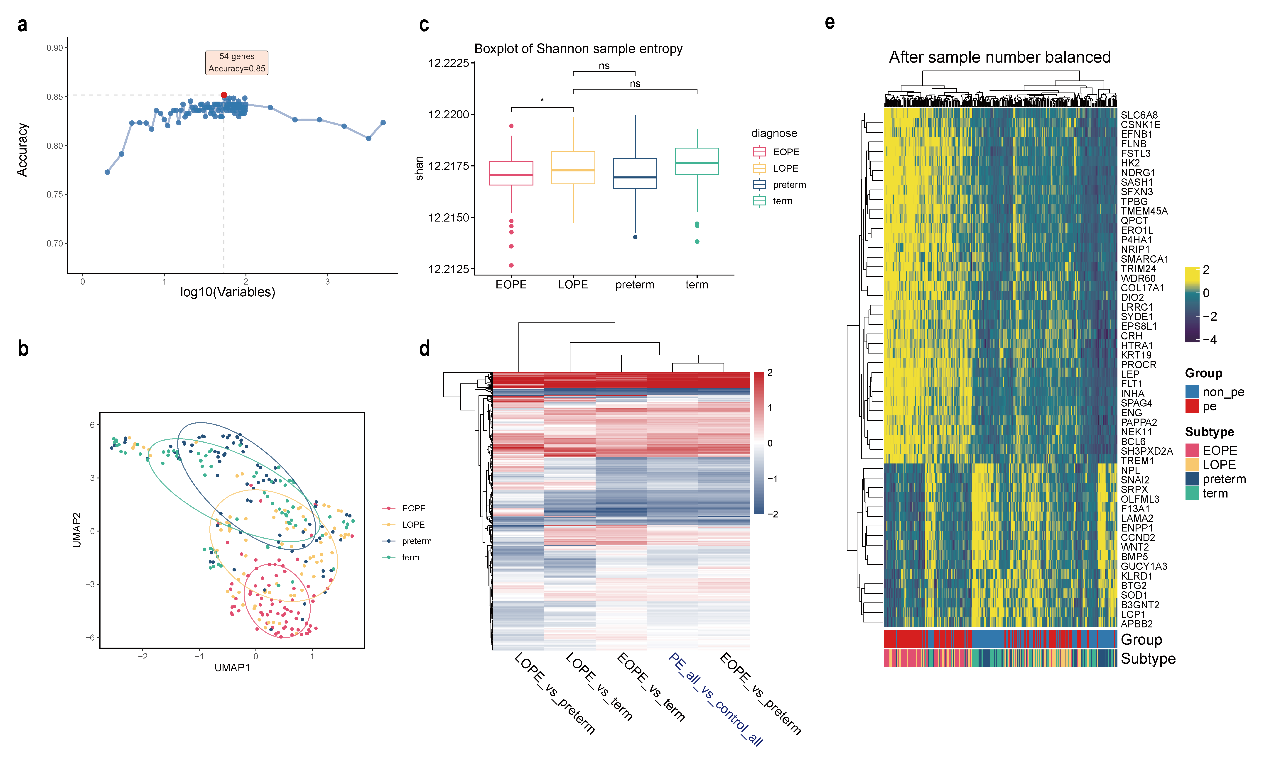


Supplementary Figure 5. Sustained high homogeneity in EOPE samples through balanced random sampling (a) The random forest recursive feature elimination (RF-RFE) model with the best subset of 54 features from the matched samples achieved 85% accuracy in classification; (b) UMAP scatter plot of the 54 PE signature genes in the matched samples; (c) Boxplot of Shannon entropy level in the matched samples; (d) Heatmap of differentially expressed genes in the matched samples, and (e) Heatmap of the expression levels of the 54 PE signature genes in the matched samples.

**Supplementary Figure6**


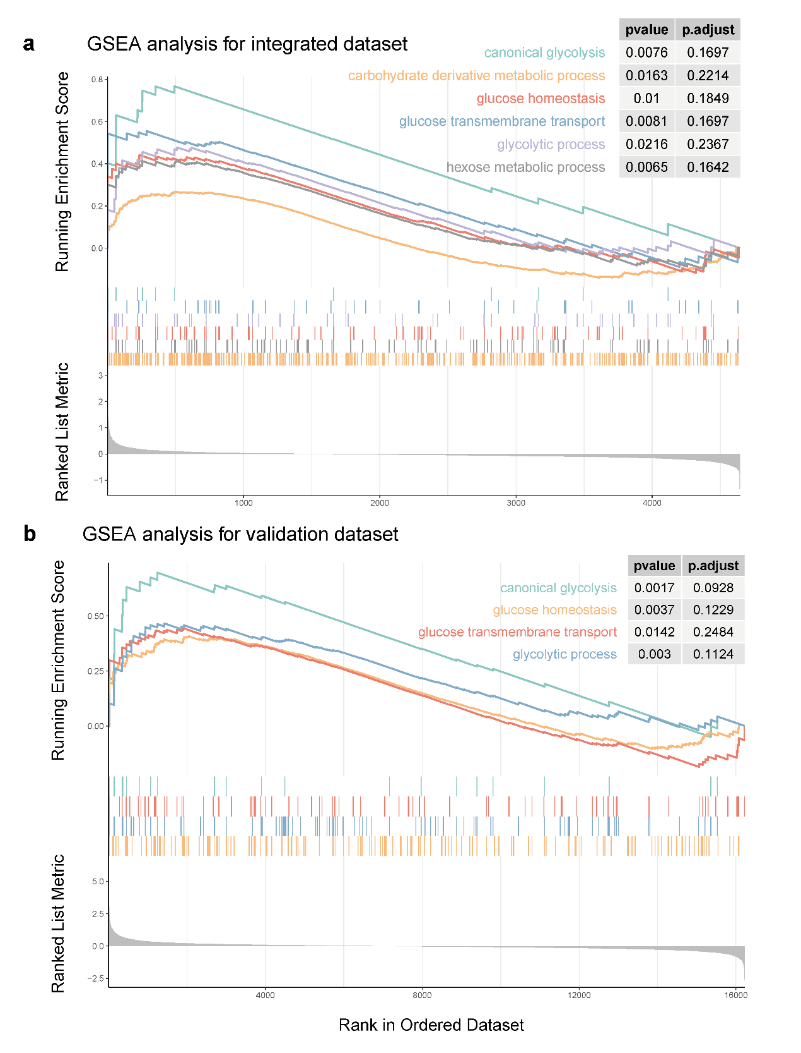


Supplementary Figure 6. GSEA analysis of glycolysis-related pathways. GSEA results of glycolysis-related pathways in the integrated dataset (a) and validation dataset (b).

**Supplementary Figure7**


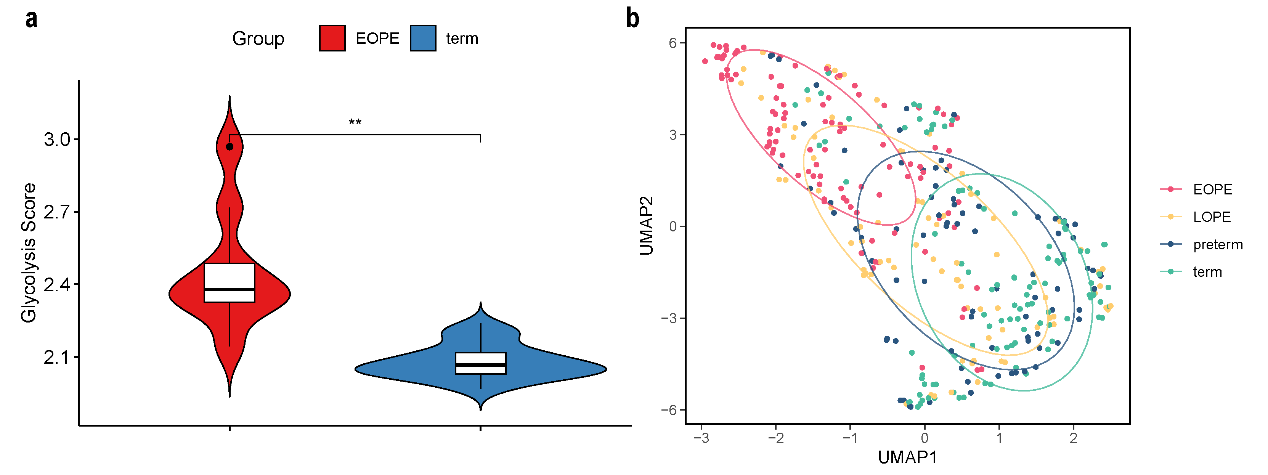


Supplementary Figure 7. Glycolysis-related genes can distinguish EOPE from other samples in both integrated and validation datasets. (a) Violin plot of glycolysis score levels in the validation dataset samples; (b) Sample umap plot of glycolysis-related genes in the integrated dataset.

**Supplementary Figure8**


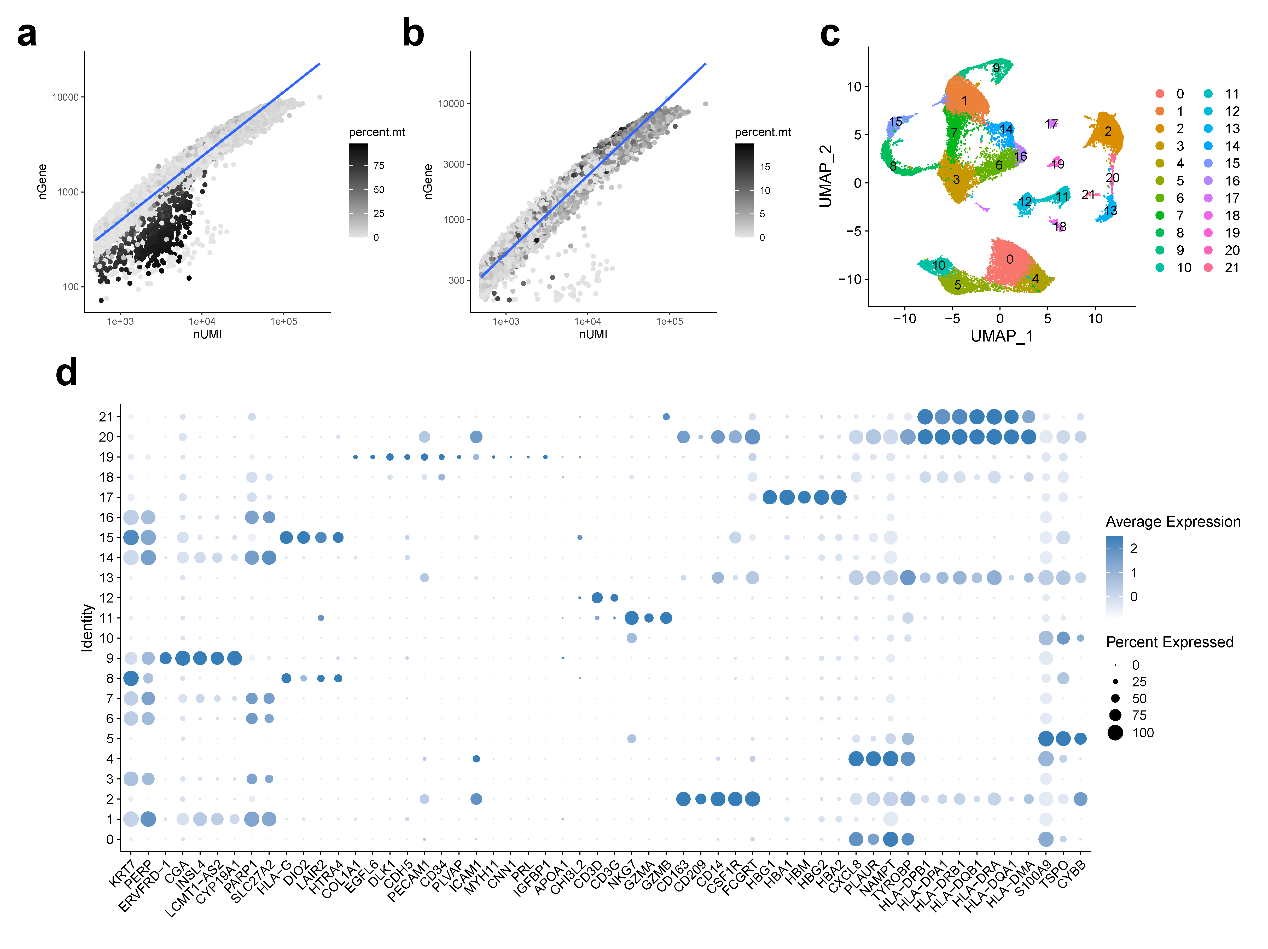


Supplementary Figure 8. Quality control, cell clustering, and cell annotation of single-cell data. Scatter plot of cells with gene numbers (nGene) and unique molecular identifiers (UMI) in raw single-cell data before (a), and after (b) quality control (QC). The grayscale of the dots represents the percentage of mitochondrial genes. (c) UMAP visualization of cell clustering after QC. (d) Bubble plot of marker gene expression for placental cell subtypes after clustering.

**Supplementary Figure9**


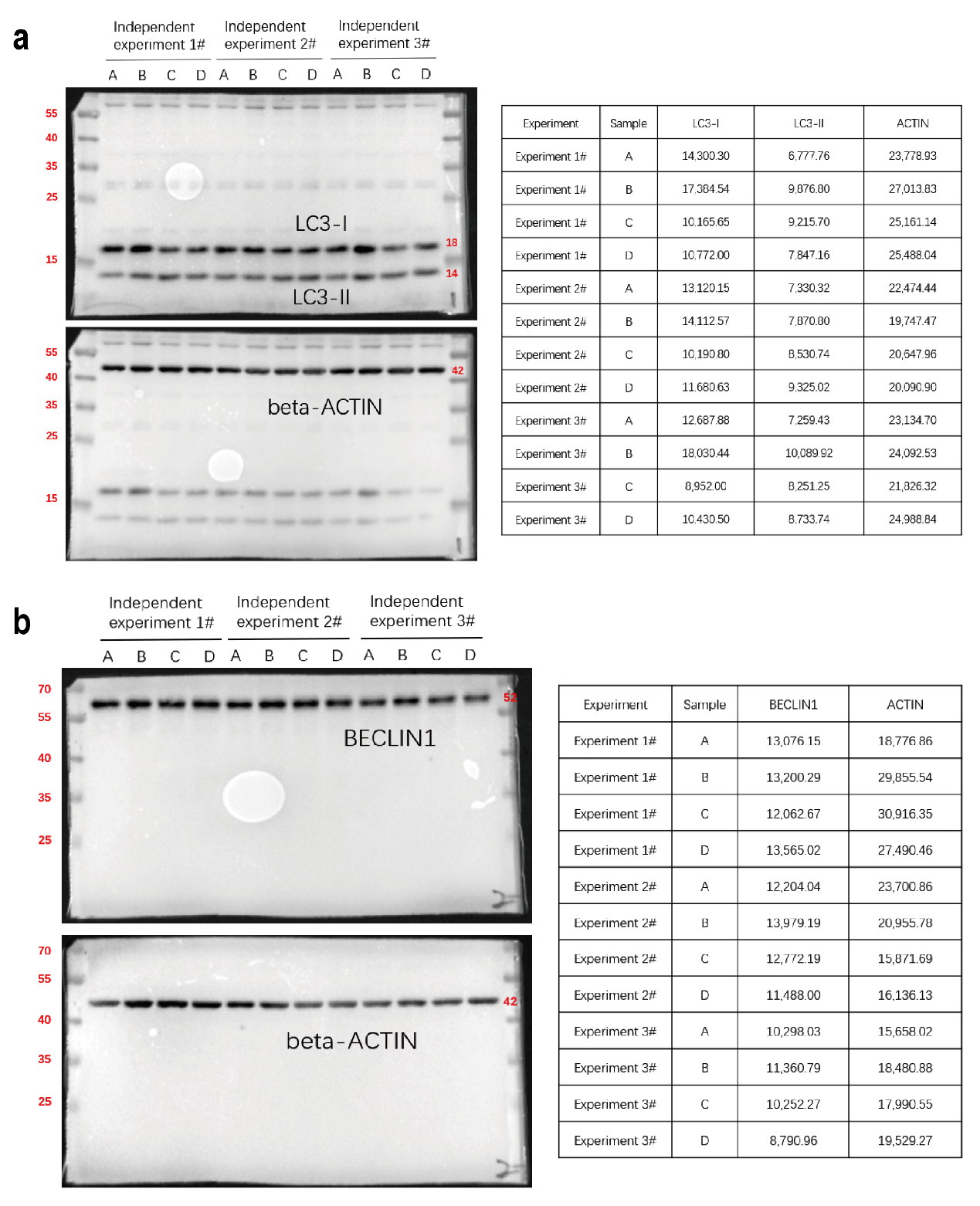


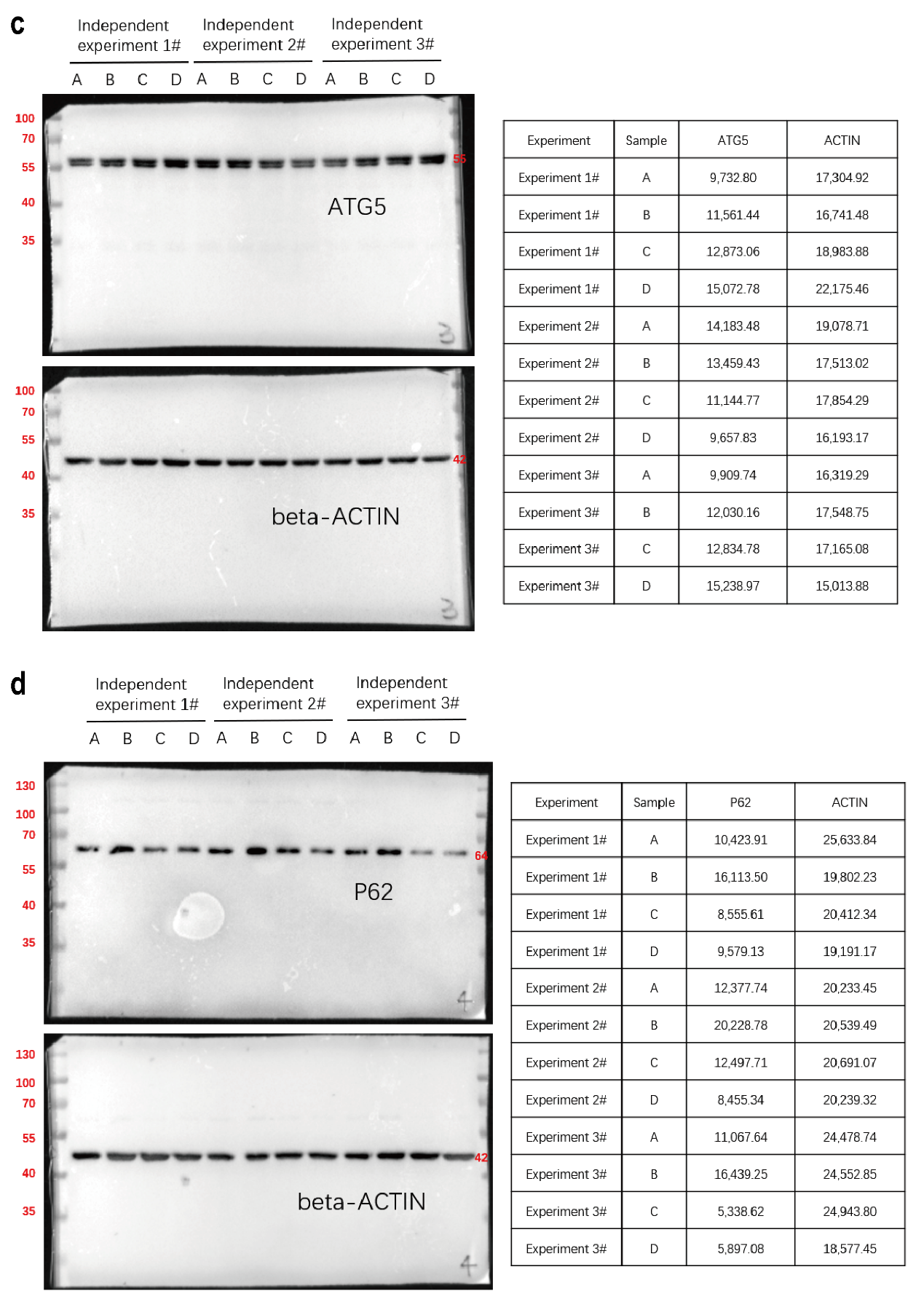


Supplementary Figure 9. Original gel images generated from western blotting. Original gel images of LC3(a), BECLIN1(b), ATG5(c), P62(d) and their gray value. A, B, C, and D represent four treatment groups: normoxia + siNC, normoxia + siHK2, hypoxia + siNC, and hypoxia + siHK2, respectively.
